# Role of the IgG4-related cholangitis autoantigen annexin A11 in cholangiocyte protection

**DOI:** 10.1016/j.jhep.2021.10.009

**Published:** 2021-10-27

**Authors:** Toni Herta, Remco Kersten, Jung-Chin Chang, Lowiek Hubers, Simei Go, Dagmar Tolenaars, Coen C. Paulusma, Michael H. Nathanson, Ronald Oude Elferink, Stan F.J. van de Graaf, Ulrich Beuers

**Affiliations:** 1 Department of Gastroenterology and Hepatology and Tytgat Institute for Liver and Intestinal Research, AGEM, Amsterdam University Medical Centers, location AMC, Amsterdam, The Netherlands;; 2 Section of Digestive Diseases, Department of Internal Medicine, Yale University School of Medicine, New Haven, USA

**Keywords:** anoctamin-1, annexin A11, autoimmunity, bicarbonate umbrella, cholangiopathy, IgG4-related systemic disease, membrane trafficking

## Abstract

**Background & Aims:**

Annexin A11 was identified as autoantigen in IgG4-related cholangitis (IRC), a B-cell driven disease. Annexin A11 modulates calcium-dependent exocytosis, a crucial mechanism for insertion of proteins into their target membranes. Human cholangiocytes form an apical ‘biliary bicarbonate umbrella’ regarded as defense against harmful hydrophobic bile acid influx. The bicarbonate secretory machinery comprises the chloride/bicarbonate exchanger AE2 and the chloride channel ANO1. We aimed to investigate the expression and function of annexin A11 in human cholangiocytes and a potential role of IgG1/IgG4-mediated autoreactivity against annexin A11 in the pathogenesis of IRC.

**Methods:**

Expression of annexin A11 in human liver was studied by immunohistochemistry and immunofluorescence. In human control and *ANXA11* knockdown H69 cholangiocytes, intracellular pH, AE2 and ANO1 surface expression, and bile acid influx were examined using ratio microspectrofluorometry, cell surface biotinylation, and 22,23-^3^H-glycochenodeoxycholic acid permeation, respectively. The localization of annexin A11-mEmerald and ANO1-mCherry was investigated by live-cell microscopy in H69 cholangiocytes after incubation with IRC patient serum containing anti-annexin A11 IgG1/IgG4-autoantibodies or disease control serum.

**Results:**

Annexin A11 was strongly expressed in human cholangiocytes, but not hepatocytes. Knockdown of *ANXA11* led to reduced plasma membrane expression of ANO1, but not AE2, alkalization of intracellular pH and uncontrolled bile acid influx. High intracellular calcium conditions led to annexin A11 membrane shift and colocalization with ANO1. Incubation with IRC patient serum inhibited annexin A11 membrane shift and reduced ANO1 surface expression.

**Conclusion:**

Cholangiocellular annexin A11 mediates apical membrane abundance of the chloride channel ANO1, thereby supporting biliary bicarbonate secretion. Insertion is inhibited by IRC patient serum containing anti-annexin A11 IgG1/IgG4-autoantibodies. Anti-annexin A11 autoantibodies may contribute to the pathogenesis of IRC by weakening the ‘biliary bicarbonate umbrella’.

## Lay summary

We previously identified annexin A11 as a specific autoantigen in immunoglobulin G4-related cholangitis (IRC), a B-cell driven disease affecting the bile ducts. Human cholangiocytes are protected against harmful hydrophobic bile acid influx by a defense mechanism referred to as the ‘biliary bicarbonate umbrella’. We found that annexin A11 is required for the formation of a robust bicarbonate umbrella. Binding of patient-derived annexin A11 autoantibodies inhibits annexin A11 function, possibly contributing to bile duct damage by weakening the biliary bicarbonate umbrella in patients with IRC.

## Graphical Abstract

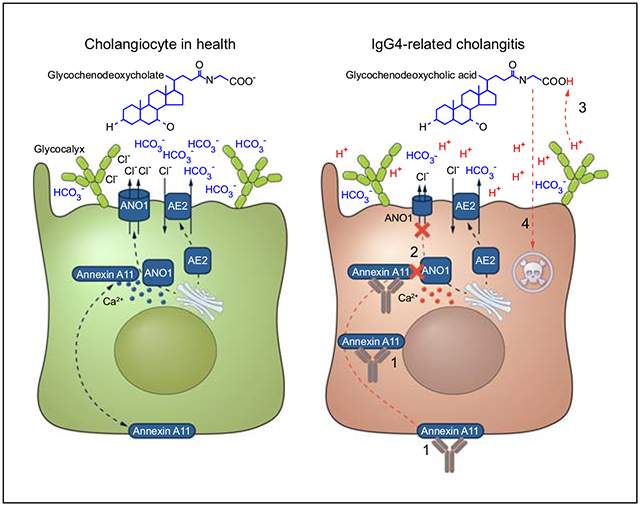

## Introduction

IgG4-related cholangitis (IRC) is the hepatobiliary manifestation of IgG4-related disease, a systemic immune-mediated disorder characterized by typical histopathological findings in affected organs and often elevated IgG4 plasma levels.^1,2^ IRC is, next to primary biliary cholangitis (PBC) and primary sclerosing cholangitis (PSC), regarded as an immune-mediated cholestatic liver disease. The pathogenesis of IRC remains poorly understood. Affinity maturated dominant IgG4^+^ B-cell receptor clones which we identified in blood and affected tissue of patients with IRC are suggestive of a B-cell driven immune reaction against a specific autoantigen.^3,4^ The disappearance of these B-cell clones in patients responding to corticosteroid treatment and their re-emergence in disease relapse underline their potential pathogenic relevance.^3,5^ Mice injected with IgG1 and IgG4 antibodies isolated from the sera of patients with IgG4-related disease developed typical organ lesions, with more destructive changes induced by IgG1 than by IgG4 when injected separately.^6^ The pathogenic activity of patient IgG1 in these mice was reduced by simultaneous injection of patient IgG4.^6^ Recently, our group identified the protein annexin A11 as the first specific autoantigen in IRC.^7^ Two epitopes on annexin A11 were recognized by both IgG4 and IgG1 plasma autoantibodies of patients with IRC but not disease controls. Notably, IgG4 blocked the binding of IgG1 to annexin A11.^7^ Together, these findings suggest that IgG4 might dampen an IgG1-driven immune response that potentially targets annexin A11 in IRC.

Annexin A11 is one of the 12 members of the human annexin superfamily.^8,9^ The protein is expressed predominantly in the cytosol and the nucleoplasm of various cell types.^10^ Annexin A11, like other annexin family members, contains 4 C-terminal Ca^2+^-sensitive membrane-binding domains, the so called ‘core region’.^8,11^ Binding of Ca^2+^ confers a positive surface charge to these domains and leads to a conformational change of the tertiary protein structure of annexin A11, facilitating the binding of cytosolic annexin A11 to negatively charged phospholipids on plasma membranes.^11,12^ Unlike the C-terminal core region, which is highly conserved among the annexins, the N-terminal low complexity region shows great variability in length and amino acid sequence.^9,13^ With 197 amino acid residues annexin A11 possesses the longest N-terminus of all known annexin family members.^9,14^ In neurons, the N-terminus of annexin A11 interacts with RNA granules while the C-terminus attaches to lysosomes, allowing for the long-distance transport of RNA granules that hitchhike on lysosomal vesicle movement.^11^

Several annexins (including annexin A1, A2, A11 and A13b) have been implicated in membrane trafficking from the endoplasmic reticulum (ER) to the apical plasma membrane.^15–18^ Annexin A11 was shown to mediate Ca^2+^-dependent vesicle trafficking from the ER to the trans-Golgi network.^18^ In pancreatic β-cells, annexin A11 is essential for an effective Ca^2+^-dependent membrane insertion of insulin granules.^19^

In cholangiocytes, Ca^2+^-dependent membrane trafficking is crucial for adequate cholangiocellular secretion and its therapeutic modulation in cholestasis.^20–22^ The insertion of membrane transporters and channels into the apical cell membrane, their site of action, is thought to be mediated by Ca^2+^-dependent proteins.^23^ The Ca^2+^-sensitive Cl^−^ channel anoctamin-1 (ANO1, also known as TMEM16A) is located on the apical cell membrane of human cholangiocytes. ANO1 mediates Cl^−^ secretion, which allows the exchange of extracellular Cl^−^ with HCO_3_^−^ by the anion exchanger 2 (AE2) on the apical surface of human cholangiocytes.^24^ This exchange is indispensable for the maintenance of a cholangiocellular defense mechanism against the toxic effects of glycine-conjugated bile acids in human bile described by us as the ‘biliary HCO_3_^−^ umbrella’.^25–27^ The biliary HCO_3_^−^ umbrella is thought to consist of a thin layer of secreted HCO_3_^−^ trapped by a dense glycocalyx on the apical cholangiocellular surface. This alkaline layer would prevent the protonation of glycine-conjugated bile salts, which, when protonated, can enter cholangiocytes in an uncontrolled fashion as bile acids and induce cellular damage.^26^ Defective apical cholangiocellular HCO_3_^−^ secretion has been proposed as an early step in the pathogenesis of PBC and PSC.^25–34^

Herein, we investigate the role of the IRC autoantigen annexin A11 in modulating cholangiocellular HCO_3_^−^ secretion by mediating the apical membrane insertion of ANO1 in a human cholangiocyte cell model. Furthermore, we study the potential relevance of IgG1-/IgG4-mediated autoreactivity against annexin A11 for the stability of the biliary bicarbonate umbrella and the pathogenesis of IRC.

## Materials and methods

Chemicals were obtained from Sigma-Aldrich unless otherwise indicated.

### Human ethics statement

The use of human liver tissue biopsies within this study was approved by the Institutional Review Board on the Protection of the Rights of Human Subjects (Yale University; HIC-1304011763). The use of patient serum samples was approved by the local medical ethical committee in Amsterdam (MEC 10/007). Participants gave written informed consent prior to inclusion in the study.

### Immunohistochemistry and immunofluorescence in human liver tissue

For immunohistochemistry, paraffin-embedded human liver specimens were cut to 4.5 μm sections using Rotary Microtome HM 340E (Thermo Scientific, Waltham, MS). Sections were stretched on glass slides and dried overnight at 37°C. After deparaffinization, antigen retrieval was performed by boiling the sections for 20 minutes at 120°C in 0.01 M sodium citrate (pH 6). Sections were blocked for 8 minutes with Ultravision protein block (Thermo Scientific, Waltham, MS) and incubated overnight with anti-human annexin A11 primary antibody (Santa Cruz, Dallas, TX) 1:200 in antibody diluent (ScyTek, Logan, UT). After incubation with BrightVision Poly-horseradish peroxidase (HRP)-anti-mouse (Avantor, Radnor, PA), sections were stained with Red AP substrate kit (Vector, Burlingame, CA) and counterstained with hematoxylin. Microscopy was performed using Olympus BX51 (Olympus Corporation, Tokyo, Japan) at a 20x objective.

For immunofluorescence, paraffin-embedded human liver tissue sections were deparaffinized and rehydrated for 40 minutes in Trilogy (Cell Marque, Rocklin, CA). Antigen retrieval was performed in 10 mM citrate buffer (pH 6) and autofluorescence was quenched for 20 minutes with 50 mM NH_4_Cl in PBS (pH 7.4). After blocking for 1 hour with 5% normal goat serum in 1% BSA/TBST (tris-buffered saline with 0.05% (w/v) Tween 20), sections were triple-incubated with anti-human annexin A11 primary antibody (Santa Cruz, Dallas, TX), anti-human ANO1 primary antibody (Abcam, Cambridge, United Kingdom) and anti-human AE2 primary antibody (Santa Cruz, Dallas, TX) 1:50 in 1% BSA/TBST overnight at 4°C. Sections were then incubated for 1 hour with the following secondary antibodies: anti-rabbit Alexa Fluor 555 (Invitrogen, Waltham, MS), anti-mouse Alexa Fluor 488 (Invitrogen, Waltham, MS) and anti-goat Alexa Fluor 647 (Invitrogen, Waltham, MS) 1:100 in 1% BSA/TBST (at room temperature, protected from light). Sections were mounted on coverglass (Thermo Scientific, Waltham, MS) with Prolong gold antifade (Invitrogen, Waltham, MS). Z-stack imaging was performed using a Leica TCS SP8 STED microscope (LEICA, Wetzlar, Germany) with a 100x/1.3 NA oil immersion objective (Zeiss, Oberkochen, Germany). Images were deconvoluted using Huygens Deconvolution Professional software (Scientific Volume Imaging, Hilversum, the Netherlands). The apical region of interest (ROI) was selected and the Pearson’s correlation coefficient^35^ was calculated using IMARIS 9.3.1 software (Oxford Instruments, Abington, UK). ImageJ 1.50i (Wayne Rasband, National Institutes of Health, Bethesda, MD) was used for histogram stretching. Human liver tissue samples were obtained from 3 different patients resected for hepatic metastases of primary colorectal carcinoma. The embedded and analyzed tissue was distant to the site of the metastases.

### Cell cultures

H69 cholangiocytes, kindly provided by Dr. Douglas Jefferson (Tufts University, Boston, MS), were cultured in a 5% CO_2_ incubator as previously described^36^ and were passaged twice per week. The culture medium contained Dulbecco’s modified Eagle’s medium/Ham’s nutrient mixture F-12 (Life Technologies Carlsbad, CA) (3:1) with 10% fetal bovine serum (Thermo Scientific, Waltham, MS) and the following supplements: 180 μM adenine, 865 nM insulin, 62.5 nM transferrin, 2 nM triiodothyronine, 1.1 μM hydrocortisone, 5.5 μM epinephrine, 1.67 nM epidermal growth factor, 37.5 U/ml penicillin, 37.5 μg/ml streptomycin, 1.75 g/L sodium bicarbonate and 20 mM 4-(2-hydroxyethyl)-1-piperazine ethanesulfonic acid-sodium hydroxide (pH 7.4). Mycoplasma contamination testing was carried out every 3 months with negative results throughout.

### Lentiviral transduction for shRNA-mediated knockdown of *ANXA11* and *ANO1* and annexin A11-eMerald overexpression

Lentiviral constructs for short hairpin RNA (shRNA)-mediated knockdown (KD) were purchased from MISSION^®^ TRC shRNA library (Sigma-Aldrich, St. Louis, MO): *ANXA11* (TRCN0000056377), *ANO1* (TCRN0000040263) and shRNA control (SHC002). The lentiviral construct for annexin A11-mEmerald overexpression was obtained from Addgene (Watertown, MA). Lentivirus was produced as previously described.^37^ Transduced H69 cholangiocytes were selected with 1 μg/ml puromycin to obtain stable KD and overexpression cell lines.

### RNA isolation, reverse-transcription and real-time quantitative PCR

Total RNA was isolated from cultured H69 cholangiocytes with TRIzol reagent (Invitrogen, Waltham, MS) and RNA concentrations were quantified by spectrophotometry at 260 nm wavelengths using Nanodrop 1000 (Thermo Scientific, Waltham, MS). 2 μg total RNA was treated with deoxyribonuclease I (Prometa, Madison, WI), and hereafter transcribed into complementary DNA (cDNA) with deoxythymidine oligomer, random hexamers, and RevertAid reverse transcriptase (Thermo Scientific, Waltham, MS). 2 μl of diluted cDNA (1:10 in water) served as template for quantitative reverse-transcription PCR (qRT-PCR) using the SensiFAST SYBR No-ROX kit (Bioline, London, United Kingdom) and a LightCycler 480 II instrument (Roche Diagnostics, Rotkreuz, Switzerland). Fluorescence values were calculated by LinRegPCR version 2013.0 (Academic Medical Center, Amsterdam, The Netherlands), and expression levels were normalized to the geomean of human *ACTB* and *36B4*. Primer sequences are listed in Table S3.

### Intracellular acidification by sodium acetate

To induce cytosolic acidification, medium was removed from H69 culture dishes and equivalent amounts of H69 culture medium supplemented with 20 mM sodium acetate or 20 mM sodium chloride (control) were added. Cells were then incubated for 10 minutes in a 5% CO_2_ incubator at 37°C. Cell surface biotinylation assays were performed as described below.

### Cell surface biotinylation assay

H69 cholangiocytes were cultured on 100×20 mm cell culture dishes (Corning, Corning, United Kingdom) until confluence. H69 monolayers were washed twice with ice-cold PBS-CM (1x PBS with 1 mM MgCl_2_, 0.5 mM CaCl_2_, pH 8) and incubated with 1 ml per dish of fresh 0.75 mg/ml NHS-ss-Biotin (Thermo Scientific, Waltham, MS) in PBS-CM for 30 minutes at 4°C on a orbital shaker (GFL, Burgwedel, Germany). After washing 2 times with PBS-CM plus 7.5 mg/ml glycine and once with PBS-CM, cells were lysed for 30 minutes in radioimmunoprecipitation assay (RIPA) lysis buffer (150 mM NaCl, 50 mM Tris pH 7.4, 5 mM EDTA, 1% Nonidet P40) supplemented with 1x protease inhibitors (Roche Diagnostics, Rotkreuz, Switzerland) for 30 minutes at 4°C on an SB3 rotator (Stuart, Staffordshire, United Kingdom). Protein amount was quantified in the lysates using the bicinchoninic acid assay (Thermo Scientific, Waltham, MS). Equal amounts of protein were incubated with neutravidin beads (Thermo Scientific, Waltham, MS) overnight at 4°C on an SB3 rotator. Beads were washed 3 times with RIPA lysis buffer and biotinylated proteins were eluted in Laemmli buffer and 0.1 M dithiothreitol for immunoblotting.

### Western blotting

After SDS-PAGE, proteins were transferred by semi-dry blotting to polyvinylidene difluoride membranes (Millipore, Burlington, VT), blocked for 90 minutes in 5% non-fat milk/TBST and probed overnight at 4°C with the respective primary antibody (list of antibodies and dilutions: see Table S1) in 5% non-fat milk/TBST. Immune complexes were detected with HRP-conjugated secondary antibodies (Table S1) and visualized using enhanced chemiluminescence detection reagent (Lumi-light, Roche Diagnostics, Rotkreuz, Switzerland) and ImageQuant LAS 4000 (GE Healthcare, Chicago, IL). Protein bands were quantified using ImageJ 1.50i (Wayne Rasband, National Institutes of Health, Bethesda, MD). Results were presented as fold changes of sham transduced H69 cells, normalized per experiment.

### Intracellular pH measurement by 2’,7’-Bis-(2-Carboxyethyl)-5-(and-6)-Carboxyfluorescein (BCECF)

H69 cholangiocytes were cultured in 96-well black plates with clear bottom (Corning, Corning, United Kingdom) until confluence. The BCECF assay was performed as described previously.^27^ Normal and chloride-free Hanks’ balanced salt solution (HBSS) (composition: see Table S2) were equilibrated overnight to 5% CO_2_ and 37°C in the incubator prior to the assay. H69 monolayers were loaded with 3 μM BCECF- acetoxymethyl ester (Molecular probes, Eugene, OR) in normal HBSS for 45 minutes at 37°C and 5% CO_2_.

### Bile acid permeation assay

H69 cholangiocytes were cultured in 24-well plates (VWR, Radnor, PA) until confluence. Monolayers were washed once with 20 mM HEPES buffered HBSS pH 7.4 (Lonza, Basel, Switzerland) and equilibrated for 30 minutes with HEPES buffered HBSS pH 7.4 at 37°C in ambient air. HBSS was removed and 200 μl per well HEPES buffered HBSS pH 7.4 with ~2,87 kBq per well 22,23-^3^H-sodium glycochenodeoxycholate (GCDC), kindly provided by Dr. Alan Hofmann (University of California, San Diego, CA), and 750 μM unlabeled sodium GCDC (Sigma-Aldrich, St. Louis, MO) was added. Bile salts were removed after 1, 4, 16 and 64 minutes. Cells were washed once with ice-cold PBS and once with 200 μl of ice-cold PBS containing 2% fatty acid-free BSA (Sigma-Aldrich, St. Louis, MO) to remove potentially membrane-bound GCDC. Cells were incubated with 50 μg/ml digitonin (Merck, Kenilworth, NJ) in ice-cold PBS to permeabilize the plasma membrane to extract the cytosolic fraction. Radio-activity in the cytosolic fraction was detected by liquid scintillation counter Tri-carb 2900TR (Perkin Elmer, Groningen, The Netherlands). Cells were then washed with ice-cold PBS and shaken in 0.05% SDS in MQ for 45 minutes on a plate shaker at room temperature. Bicinchoninic acid assays were performed on both the cytosolic and membrane fraction to correct 22,23-^3^H-GCDC bile acid permeation values for total protein content per well. Values were normalized to 1 minute 22,23-^3^H-GCDC bile acid permeation of sham H69 cells.

### Colocalization of annexin A11-mEmerald and ANO1-mCherry

Annexin A11-mEmerald overexpressing H69 cholangiocytes were seeded onto 8-well chambered coverglass (Thermo Scientific, Waltham, MS) and cultured for 96 hours until confluence. 24 hours after seeding, cells were transfected with ANO1-mCherry,^38^ kindly provided by Dr. Lily Yeh Jan (University of California, San Diego, CA). Live-cell confocal microscopy was performed in Leibowitz imaging medium without phenol red (Thermo Scientific, Waltham, MS) at 37°C on a Leica TCS SP8-SMD microscope (LEICA, Wetzlar, Germany). 10 μM ionomycin (Abcam, Cambridge, United Kingdom) in Leibowitz imaging medium was added to increase intracellular Ca^2+^ levels. Cells were imaged at 15–30 second intervals for a duration of 15 minutes at 37°C in ambient air. 2D colocalization analysis was performed using Huygens Deconvolution Professional software (Scientific Volume Imaging, Hilversum, The Netherlands). Cells that responded to ionomycin treatment (defined as annexin A11-mEmerald localization shift) and expressed ANO1-mCherry were selected as ROI for the calculation of Pearson’s correlation coefficient (using Costes threshold approach^39^).

### Prediction of epitope binding sites

The open-source software ElliPro^40^ was used to predict epitope binding sites on annexin A11 for the anti-annexin A11 autoantibodies found in the serum of patients with IRC.^7^ The crystallized structure of annexin A11 in *Rattus norvegicus* (which shares 95.73% homology with annexin A11 in *Homo sapiens*) was used as the input. A minimum score of 0.5 (protrusion index) and a maximum distance of 6 Ångström were used as input parameters. Identified discontinuous epitopes (in orange) and calcium binding sites (in cyan) were then depicted in the crystal structure by using the open-source software Jmol version 14.

### Annexin A11-mEmerald localization shift after incubation with patient serum

Annexin A11-mEmerald overexpressing H69 cholangiocytes were transiently transfected with the fluorescent Ca^2+^- indicator R-GECO1 (Addgene, Watertown, MA) and incubated for 3 days in H69 culture medium without fetal bovine serum that was supplemented with 20% serum from 3 patients with PSC or IRC. Medium including patient serum was refreshed daily. Serum treatment was carried out in a blinded fashion. Patient sera had been collected in a previous study.^7^ The patient with IRC fulfilled the diagnostic criteria for the diagnosis of IgG4-related disease (HISORt criteria), as defined in.^2,41^ The 2 patients with PSC were diagnosed according to the 2009 European Association for the Study of the Liver clinical practice guidelines on the management of cholestatic liver diseases.^42^ Anti-annexin A11 autoantibodies were identified in patient sera as specified.^7^ Patient characteristics and laboratory parameters are shown in Table 1. Live-cell confocal microscopy was performed in Leibowitz imaging medium without phenol red (Thermo Scientific, Waltham, MS) using a Leica TCS SP8-SMD microscope (LEICA, Wetzlar, Germany). 50 μM ionomycin (Abcam, Cambridge, United Kingdom) in Leibowitz imaging medium was added to increase intracellular Ca^2+^ levels. Cells were imaged at 15–30 second intervals for a duration of 15 minutes at 37°C in ambient air. Annexin A11 localization shift was quantified by counting the number of cells that demonstrated a shift from diffuse intracellular annexin A11-mEmerald localization to a membrane localization pattern. Maximum fold-change in R-GECO1 fluorescence intensity was quantified in ROIs using ImageJ 1.50i (Wayne Rasband, National Institutes of Health, Bethesda, MD).

### ANO1-mCherry plasma membrane localization after incubation with patient serum

H69 cholangiocytes were seeded in 8-well chambered coverglass and incubated for 6 days in H69 culture medium without fetal bovine serum that was supplemented with 20% serum of patients with PSC or IRC. Medium including patient serum was refreshed daily. Serum treatment was carried out in a blinded fashion. At day 4 after seeding, cells were transiently transfected with ANO1-mCherry. At day 6 after seeding, H69 medium was changed for Leibowitz imaging medium without phenol red (Thermo Scientific, Waltham, MS) supplemented with 20% of the respective patient serum. In addition, CellMask Green (Thermo Scientific, Waltham, MS) was added to the imaging medium to stain the plasma membrane. Microscopy was performed using a Leica TCS SP8-SMD microscope (LEICA, Wetzlar, Germany). Semiquantitative scoring of ANO1-mCherry plasma membrane staining was done separately in a blinded fashion by 3 researchers. Per ANO1-mCherry transfected cell, the ANO1-mCherry localization pattern was categorized as ‘strong membrane localization’, ‘light membrane localization’, or ‘no membrane localization’.

### Statistical analysis

Data are presented as medians with interquartile range unless otherwise indicated. Statistical analysis was performed with GraphPad Prism 6 (GraphPad, Ja Jolla, USA). Results of 2 groups were compared by Mann-Whitney non-parametric test, two-way ANOVA or paired or unpaired *t* test where appropriate, as specified in the Fig. legends. *P* values <0.05 were considered statistically significant.

## Results

### Annexin A11 is detected in the cytoplasm and on the apical and basolateral surface of human cholangiocytes

**Fig. 1.**
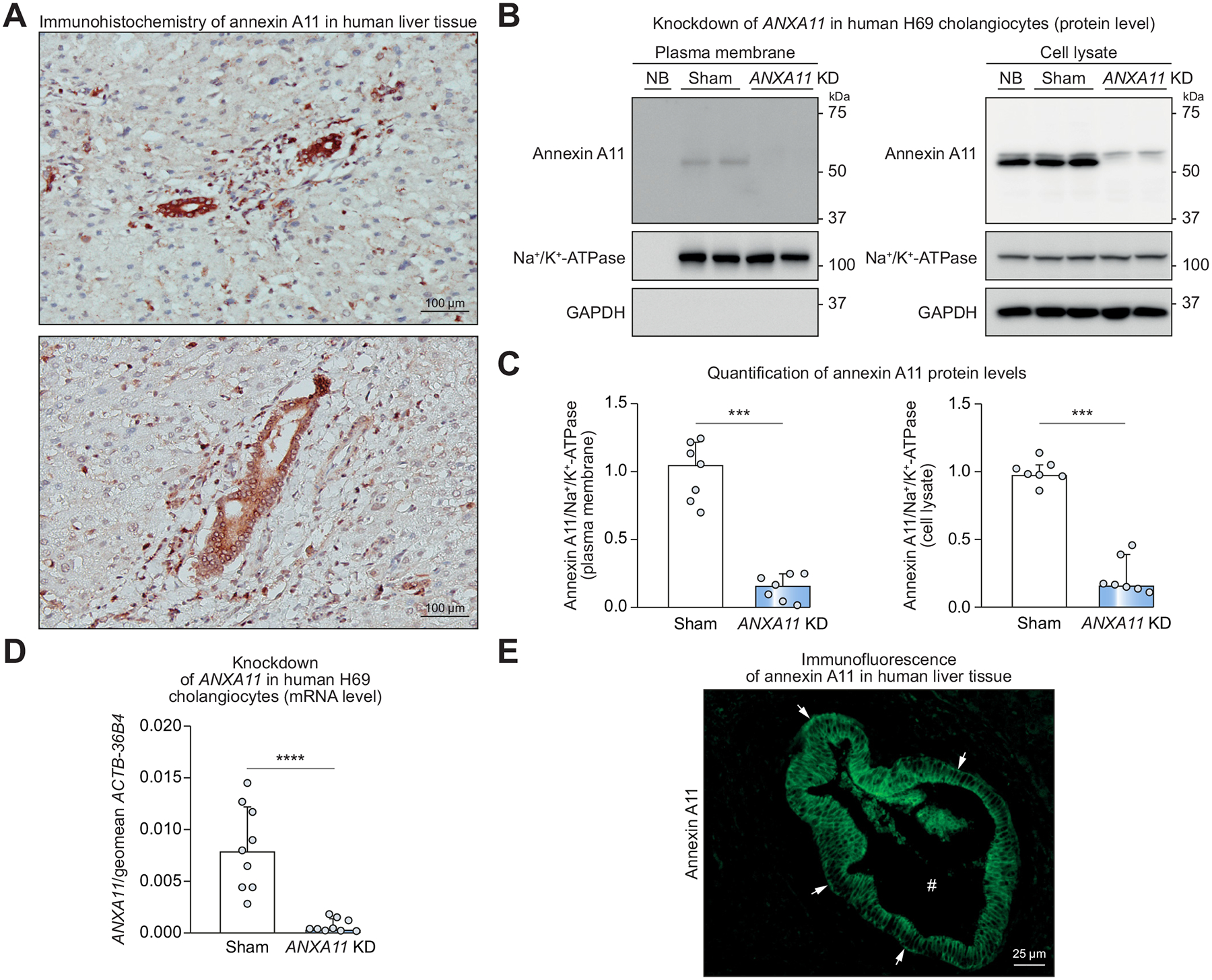
Annexin A11 is expressed in the cytoplasm and the apical and basolateral plasma membrane of human cholangiocytes. (A) Immunohistochemistry staining for annexin A11 in human liver tissue. (B) Cell surface biotinylation assay followed by immunoblotting for annexin A11 (observed molecular weight: 56 kDa) in sham and *ANXA11* KD H69 cholangiocytes. Na^+^/K^+^ ATPase is used as loading control, GAPDH serves as proof for adequate separation of biotinylated plasma membrane proteins (no GAPDH) from intracellular proteins in the cell lysate. (C) Quantification of annexin A11 protein levels (7 cell samples of n = 3 independent experiments). (D) *ANXA11* gene expression level in sham and *ANXA11* KD H69 cholangiocytes (9 cell samples of n = 3 independent experiments). (E) Immunofluorescence staining for annexin A11 in human liver tissue. ▶ basolateral cholangiocyte cell membrane, # bile duct lumen. Data are presented as median with interquartile range. Levels of significance: ***p <0.001, ****p <0.0001 (Mann-Whitney *U* test (C), unpaired *t* test (D)). KD, knockdown; NB, non-biotin control of sham H69 cholangiocytes.

To assess whether annexin A11 is expressed in cholangiocytes, the cell type targeted in IRC, paraffin-embedded human liver tissue was stained for annexin A11 using immunohistochemistry. The specimen was obtained from a patient without clinical, serological or histopathological signs of immune-mediated cholangiopathy. Staining revealed prominent expression of annexin A11 in cholangiocytes and weak expression in hepatocytes (Fig. 1A). To confirm this observation, expression of annexin A11 was analyzed in primary mouse cholangiocytes and hepatocytes using qRT-PCR. Again, annexin A11 was strongly expressed in cholangiocytes, but only weakly in hepatocytes (Fig. S1).

To further investigate the localization of annexin A11 in cholangiocytes, human SV40 (simian virus 40)-transformed cholangiocytes (H69 cholangiocytes) were transduced with shRNA against *ANXA11*, the coding gene of annexin A11, or non-targeting shRNA (sham), and surface biotinylation followed by immunoblotting was performed. Annexin A11 was detected in the total cell lysate and on the outer leaflet of the plasma membrane (surface) of sham H69 cells (Fig. 1B,C). *ANXA11* KD strongly reduced annexin A11 protein levels in the whole cell lysate and on the cell surface (Fig. 1C). Of note, *ANXA11* mRNA levels were >95 % downregulated in *ANXA11* KD compared to sham transduced H69 cells (Fig. 1D).

Immunofluorescence of bile ducts in paraffin-embedded human liver tissue showed annexin A11 localization in the cytosol and on the apical (#) and basolateral (▶) plasma membrane of human cholangiocytes (Fig. 1E). Thus, annexin A11 is present in cholangiocytes, the cell type targeted in IRC, and is expressed within the cytosol and on the apical and basolateral plasma membrane.

### Intracellular acidification increases annexin A11 surface expression while depleted annexin A11 surface expression results in intracellular alkalinization in H69 cholangiocytes

**Fig. 2.**
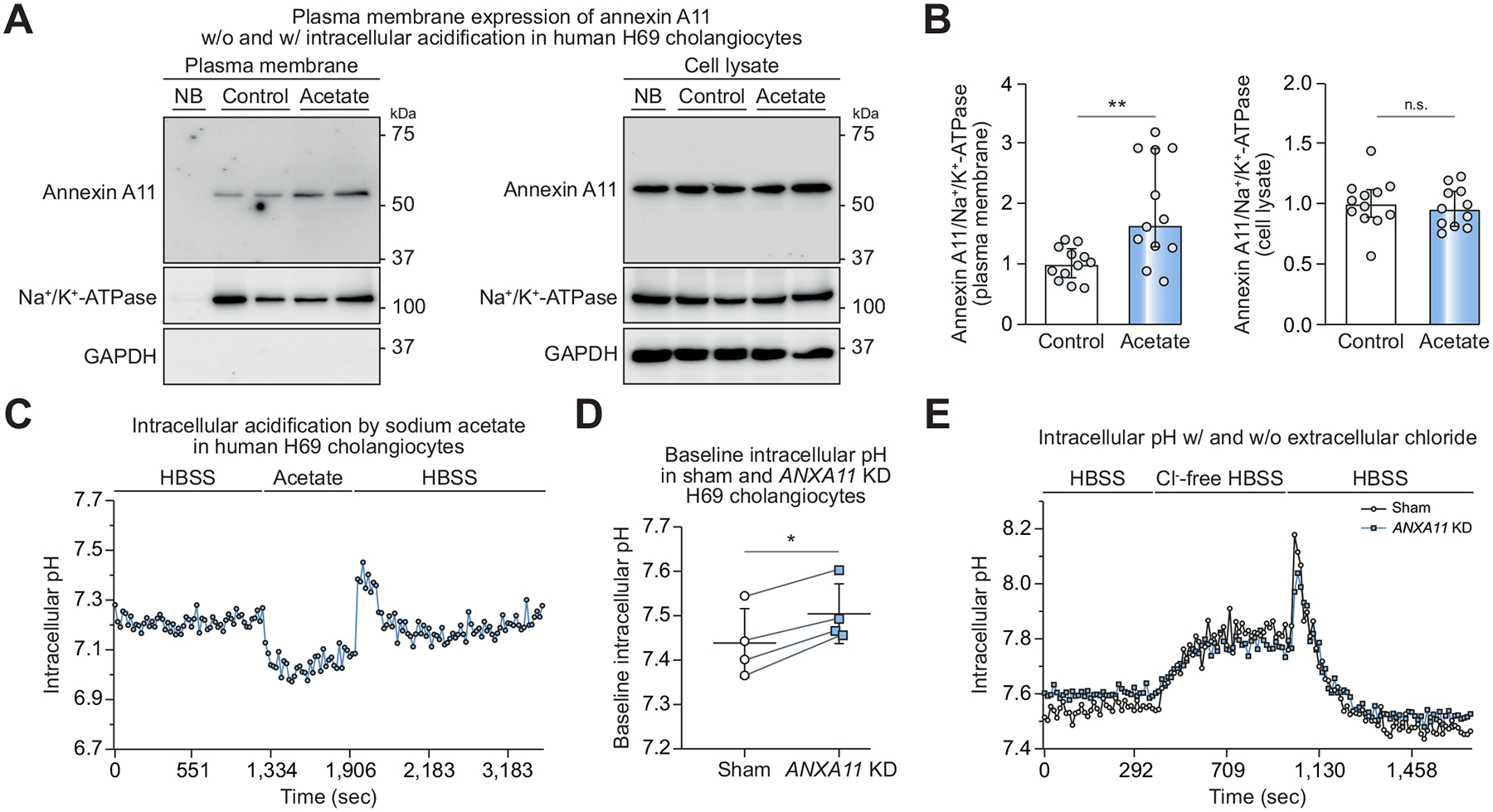
Plasma membrane expression of annexin A11 is pH-sensitive in H69 cholangiocytes. (A) Cell surface biotinylation assay followed by immunoblotting for annexin A11 in sham H69 cholangiocytes after treatment with 20 mM sodium chloride (control) or sodium acetate (Acetate) for 10 minutes. Na^+^/K^+^ ATPase is used as loading control, GAPDH serves as proof for adequate separation of biotinylated plasma membrane proteins (no GAPDH) from total cell lysate. (B) Quantification of protein levels (12 cell samples of n = 6 independent experiments). (C) Intracellular pH tracing in sham H69 cholangiocytes without (w/o) and with (w/) 20 mM sodium acetate treatment. (D) Baseline intracellular pH of sham and *ANXA11* KD H69 cholangiocytes (n = 4 independent experiments). (E) Representative intracellular pH tracing of sham and *ANXA11* KD H69 cholangiocytes in HBSS and Cl^−^-free HBSS. Data are presented as median with interquartile range (B, D) or average of duplicate (C, E). Levels of significance: *p <0.05, **p <0.01 (Mann-Whitney *U* test (B), paired t-test (D)). HBSS, Hanks’ balanced salt solution; KD, knockdown; NB, non-biotin control of sham H69 cholangiocytes.

As surface localization of homologous annexins depends on their protonation state and thus on cytosolic pH,^43,44^ cells were treated with sodium acetate to induce acute cytosolic acidification before surface biotinylation. While the total amount of annexin A11 in cell lysates did not differ between untreated (control) and treated (acetate) cells, average annexin A11 levels at the cell surface were doubled in acetate-treated cholangiocytes compared to control cholangiocytes (Fig. 2A, B). 20 mM sodium acetate, as applied in our experiments, lowered the cytosolic pH by ~0.3 in H69 cholangiocytes (Fig. 2C).

Given this effect of intracellular pH on surface expression of annexin A11, we also assessed a potential effect of total annexin A11 expression on the intracellular pH in H69 cholangiocytes using the fluorescent probe BCECF. *ANXA11* KD H69 cholangiocytes showed a more alkaline intracellular pH compared to control cells (Fig. 2D). Intracellular pH is tightly regulated in cholangiocytes and depends on intracellular HCO_3_^−^ levels, a major component of the intracellular buffer apparatus.^45^ HCO_3_^−^ is exported by AE2 on the apical surface of human cholangiocytes in exchange for extracellular Cl^−^ that is mainly provided by ANO1.^24–26^ After baseline pH measurement in normal HBSS (showing again a more alkaline intracellular pH in *ANXA11* KD H69 cells), Cl^−^ was removed from the cell supernatant to reverse the activity of the Cl^−^/HCO_3_^−^ exchanger AE2 and to diminish substrate supply for the Cl^−^ channel ANO1. Consequently, the intracellular pH of sham and *ANXA11* KD H69 cholangiocytes rose to a comparable level. Subsequent re-exposure to Cl^−^ containing HBSS reduced the intracellular pH of sham and *ANXA11* KD cells to their respective baseline values (Fig. 2E). In conclusion, annexin A11 is closely involved in regulation of intracellular pH in human H69 cholangiocytes. While surface expression of annexin A11 is increased at low intracellular pH, the more alkaline intracellular pH in *ANXA11* KD H69 cholangiocytes might indicate intracellular HCO_3_^−^ retention, potentially as a result of impaired Cl^−^-dependent HCO_3_^−^ export.

### Annexin A11 is crucial for robust ANO1, but not AE2 membrane localization and clusters in putative apical plasma membrane insertion sites in human cholangiocytes

**Fig. 3.**
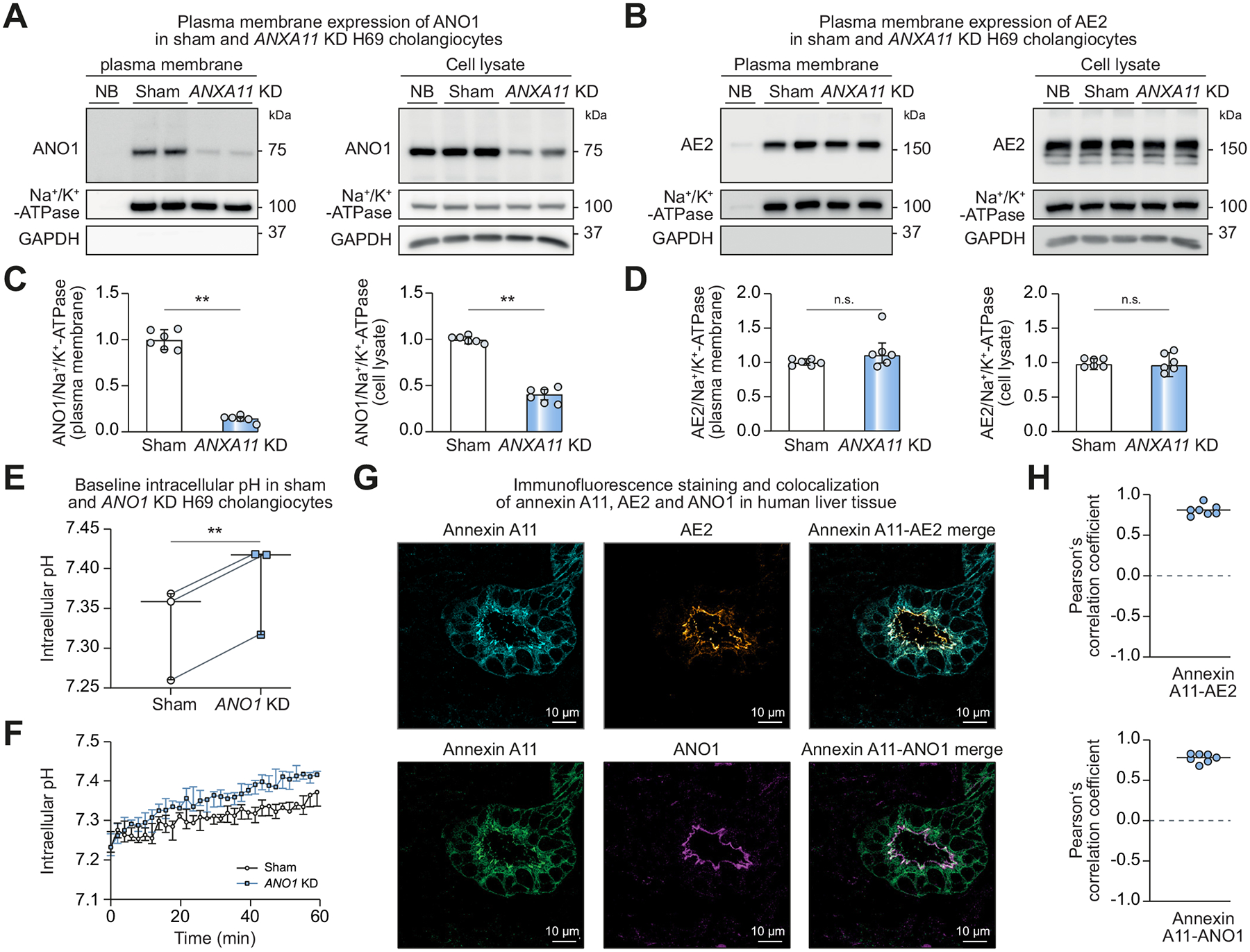
Annexin A11 is required for ANO1, but not AE2 membrane localization in human cholangiocytes. Cell surface biotinylation assay followed by immunoblotting for (A) ANO1 (observed molecular weight: 75 kDa) or (B) AE2 (observed molecular weight: 150 kDa) in sham and *ANXA11* KD H69 cholangiocytes. Na^+^/K^+^ ATPase is used as loading control, GAPDH serves as proof for adequate separation of biotinylated plasma membrane proteins (no GAPDH) from total cell lysate. (C, D) Quantification of protein levels (6 cell samples of n = 3 independent experiments). (E) Baseline intracellular pH of sham and *ANO1* KD H69 cholangiocytes (n = 3 independent experiments). (F) Representative full trace of baseline intracellular pH in sham and *ANO1* KD H69 cholangiocytes. (G) Immunofluorescence staining for annexin A11, AE2 and ANO1 in human liver. (H) Pearson’s correlation coefficients (7 bile ducts in n = 3 livers). Data are presented as median with interquartile range. Levels of significance: **p <0.01, n.s. not significant (Mann-Whitney *U* test (C, D), paired *t* test (E)). KD, knockdown; NB, non-biotin control of sham H69 cholangiocytes.

As several annexins including annexin A11 mediate membrane trafficking of proteins from the ER to the plasma membrane,^15–18^ we investigated the potential role of annexin A11 in mediating AE2 and ANO1 plasma membrane localization to further elucidate the mechanism behind the impaired Cl^−^-dependent HCO_3_^−^ export in *ANXA11* KD H69 cholangiocytes. Surface biotinylation followed by immunoblotting in sham and *ANXA11* KD H69 cells revealed that AE2 density at the plasma membrane was unchanged (Fig. 3B, D) while ANO1 was reduced by ~85% in *ANXA11* KD compared to sham H69 cholangiocytes (Fig. 3A, C). In whole cell lysates, AE2 was again unchanged while ANO1 protein levels were reduced by ~60% in *ANXA11* KD H69 cells (Fig. 3C, D).

To confirm that reduced expression of ANO1 results in intracellular alkalinization, H69 cholangiocytes were transduced with shRNA against *ANO1*, the coding gene of ANO1, or non-targeting shRNA (sham) (Fig. S2), and intracellular pH was determined. Intracellular pH was more alkaline in *ANO1* KD H69 cells compared to the corresponding sham cells (Fig. 3E, F).

As a potential regulator for plasma membrane targeting, annexin A11 likely colocalizes with ANO1 in human cholangiocytes. To investigate colocalization, paraffin-embedded human liver tissue obtained from patients after liver resections for colorectal metastases without clinical, serological or histopathological signs of immune-mediated cholangiopathy were triple-stained for annexin A11, ANO1 and AE2 using immunofluorescence. In line with the immunohistochemical staining depicted in Fig. 1, annexin A11 signal was detected in the cytoplasm and on the apical and basolateral plasma membrane of cholangiocytes (but not the surrounding hepatocytes), with predominant staining in the apical cholangiocyte plasma membrane region. ANO1 and AE2 showed strict apical cholangiocyte membrane staining (Fig. 3G). To assess the extent of colocalization between annexin A11, ANO1 and AE2, the apical bile duct region was selected as the ROI (Fig. S3). Colocalization of annexin A11 with ANO1 and AE2 in the ROI was evaluated using IMARIS imaging software. The Pearson’s correlation coefficient was 0.78 for the colocalization of annexin A11 and ANO1, and 0.81 for annexin A11 and AE2 (Fig. 3H). Thus, the degree of colocalization between annexin A11 and ANO1 and annexin A11 and AE2 in the apical membrane region of human cholangiocytes is strong.^46^ As plasma membrane insertion sites are often shared between different channels and transporters,^47,48^ we conclude that annexin A11 localizes to putative apical plasma membrane insertion sites in human cholangiocytes, where it mediates a late exocytotic step in ANO1, but not AE2 membrane trafficking.

### Colocalization of annexin A11 and ANO1 depends on intracellular free Ca^2+^ levels in H69 cholangiocytes

**Fig. 4.**
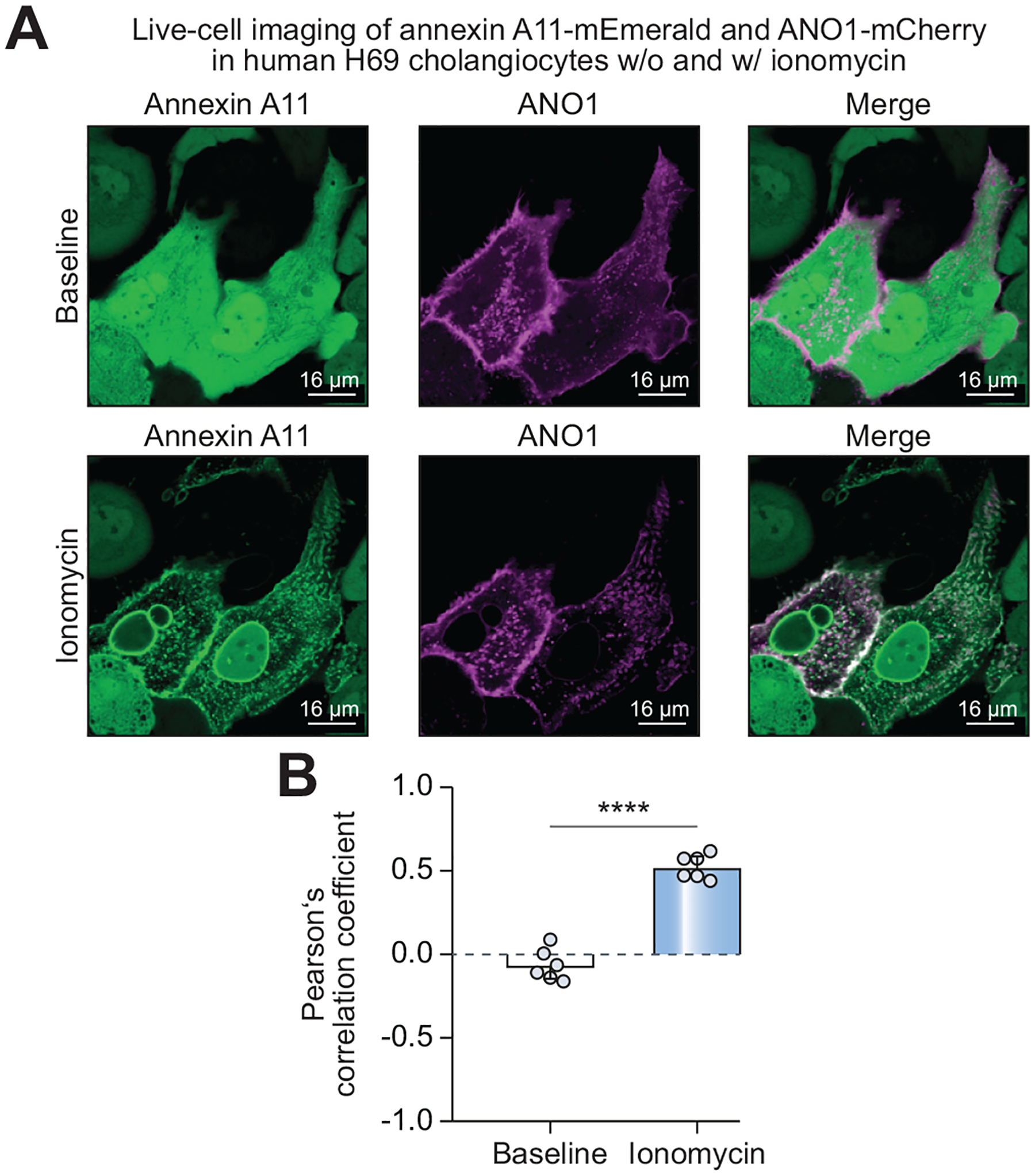
Colocalization of annexin A11 and ANO1 is stimulated by elevated intracellular free Ca^2+^ levels in H69 cholangiocytes. (A) Live-cell imaging of ionomycin treated (10 μM for 15 minutes) annexin A11-mEmerald-over-expressing and ANO1-mCherry-transfected H69 cholangiocytes. (B) Pearson’s correlation coefficient in the region of interest (defined as cells showing annexin A11-mEmerald localization shift and ANO1-mCherry expression) (6 regions of interest of n = 3 independent experiments). Data are presented as median with interquartile range. Level of significance: ****p <0.0001 (unpaired *t* test).

Insertion of membrane channels such as ANO1 into the apical cell membrane in general and annexin A11-mediated membrane insertion of vesicles in particular depend on intracellular free Ca^2+^ levels.^19–21,23^ To assess Ca^2+^-dependency of annexin A11 colocalization with ANO1, H69 cholangiocytes were transduced with fluorescently labelled annexin A11 (mEmerald-tagged overexpression construct), and transiently transfected with fluorescently labelled ANO1-mCherry. Intracellular free Ca^2+^ was increased using the Ca^2+^ ionophore ionomycin and intracellular localization of annexin A11-mEmerald and ANO1-mCherry was analyzed by live-cell microscopy (Fig. 4A). At baseline (before treatment with ionomycin) annexin A11-mEmerald showed a diffuse localization throughout the cytosol and nucleus, while ANO1-mCherry mainly localized at the plasma membrane and in an ER, trans-Golgi network and vesicular-like pattern. After treatment with ionomycin, annexin A11-mEmerald strongly shifted to membranes (plasma membrane, nuclear membrane, vesicular membrane) and colocalized with ANO1-mCherry at the plasma membrane and in vesicular-like structures in the cytosol (Suppl. Video 1; Fig. 4A, B). In conclusion, colocalization of annexin A11 and ANO1 in human cholangiocytes at the plasma membrane is Ca^2+^-dependent.

### Anti-annexin A11 autoantibodies of patients with IRC inhibit the Ca^2+^-dependent membrane shift of annexin A11 and plasma membrane localization of ANO1 in H69 cholangiocytes

**Fig. 5.**
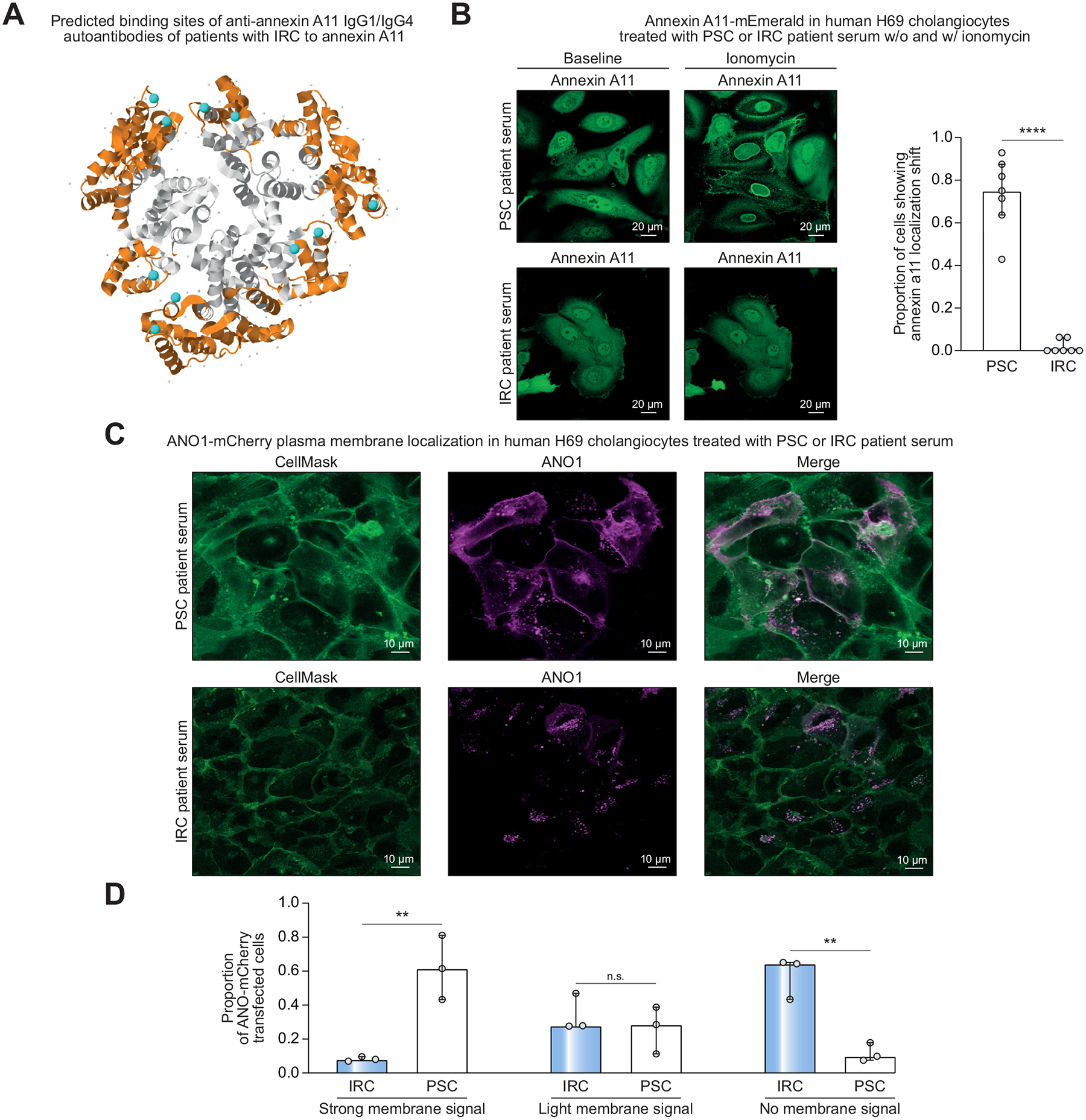
Serum of a patient with IRC containing anti-annexin A11 IgG1/IgG4-autoantibodies, but not of patients with PSC, inhibits the Ca^2+^-dependent membrane shift of annexin A11 and reduces plasma membrane localization of ANO1. (A) IgG1/IgG4-autoantibody binding sites on annexin A11 (orange) and Ca^2+^-binding domains (cyan) as predicted using the ElliPro software tool. (B) Ionomycin-treated (50 μM for 15 minutes) annexin A11-mEmerald-overexpressing H69 cholangiocytes after 3 days of incubation with 20% PSC or IRC patient serum. Proportion of cells showing annexin A11-mEmerald localization shift (7 confocal pictures of n = 3 independent experiments). (C) ANO1-mCherry plasma membrane localization in H69 cholangiocytes after 6 days of incubation with 20% PSC or IRC patient serum. (D) Semiquantitative scoring of ANO1-mCherry plasma membrane localization (1 scorer, n = 3 independent experiments; see also Fig. S5 for scorer 2 and 3). Data are presented as median with interquartile range. Level of significance: **p <0.01, ****p <0.0001 (unpaired *t* test (B), two-way ANOVA (D)). IRC, IgG4-related cholangitis; PSC, primary sclerosing cholangitis.

To investigate a potential role of anti-annexin A11 autoantibodies in patients with IRC, IgG1/IgG4-autoantibody binding sites (in orange) on annexin A11 were predicted using the Elli-Pro software tool. Prediction revealed a close proximity or containment of Ca^2+^ binding domains (in cyan) (Fig. 5A). Therefore, we hypothesized that binding of anti-annexin A11 autoantibodies could potentially block these Ca^2+^ binding domains and inhibit the Ca^2+^-dependent localization shift of annexin A11 in human cholangiocytes. Thus, annexin A11-mEmerald overexpressing H69 cholangiocytes were incubated in culture medium containing 20% serum of patients with PSC (without anti-annexin A11 autoantibodies, disease control) or 20% serum of a patient with IRC (containing anti-annexin A11 IgG1/IgG4 autoantibodies). After 3 days of incubation, intracellular free Ca^2+^ levels were increased using the Ca^2+^ ionophore ionomycin and intracellular localization of annexin A11-mEmerald was analyzed using live-cell microscopy (Fig. 5B). As a positive control for the ionomycin-induced Ca^2+^ release, the Ca^2+^ indicator R-GECO1 was transiently transfected into the annexin A11-mEmerald overexpressing H69 cholangiocytes and fold-change of R-GECO1 maximum fluorescence intensity was quantified (Fig. S4). Treatment with ionomycin induced a strong membrane shift of annexin A11-mEmerald in the vast majority of H69 cholangiocytes that were incubated with PSC patient serum (Fig. 5B). A minimal number of cells demonstrated a membrane shift of annexin A11-mEmerald after incubation with IRC patient serum (Fig. 5B). Fold-change of R-GECO1 fluorescence did not differ in H69 cells incubated with PSC and IRC serum, indicating a comparable increase of intracellular free Ca^2+^ (Fig. S4).

Ca^2+^-dependent plasma membrane shift of annexin A11 is crucial to exert its function.^11^ H69 cholangiocytes were transiently transfected with fluorescently labelled ANO1-mCherry and again incubated in culture medium containing 20% serum from patients with PSC (without anti-annexin A11 autoantibodies) or IRC (containing anti-annexin A11 autoantibodies). After 6 days of incubation, plasma membranes were stained with CellMask Green and the plasma membrane localization of ANO1-mCherry was analyzed using immunofluorescence microscopy (Fig. 5C). Semiquantitative scoring revealed a strong ANO1-mCherry plasma membrane localization after incubation with PSC patient serum. In contrast, the majority of cells showed no or minimal plasma membrane localization of ANO1-mCherry after incubation with IRC patient serum (Fig. 5D and Fig. S5). In conclusion, serum of patients with IRC containing anti-annexin A11 IgG1/IgG4-autoantibodies inhibits the Ca^2+^-dependent plasma membrane localization shift of annexin A11 and, thereby, plasma membrane targeting of ANO1 by annexin A11 in human cholangiocytes.

### Knockdown of annexin A11 and ANO1 expression increases plasma membrane permeability for glycine-conjugated bile acids in H69 cholangiocytes

**Fig. 6.**
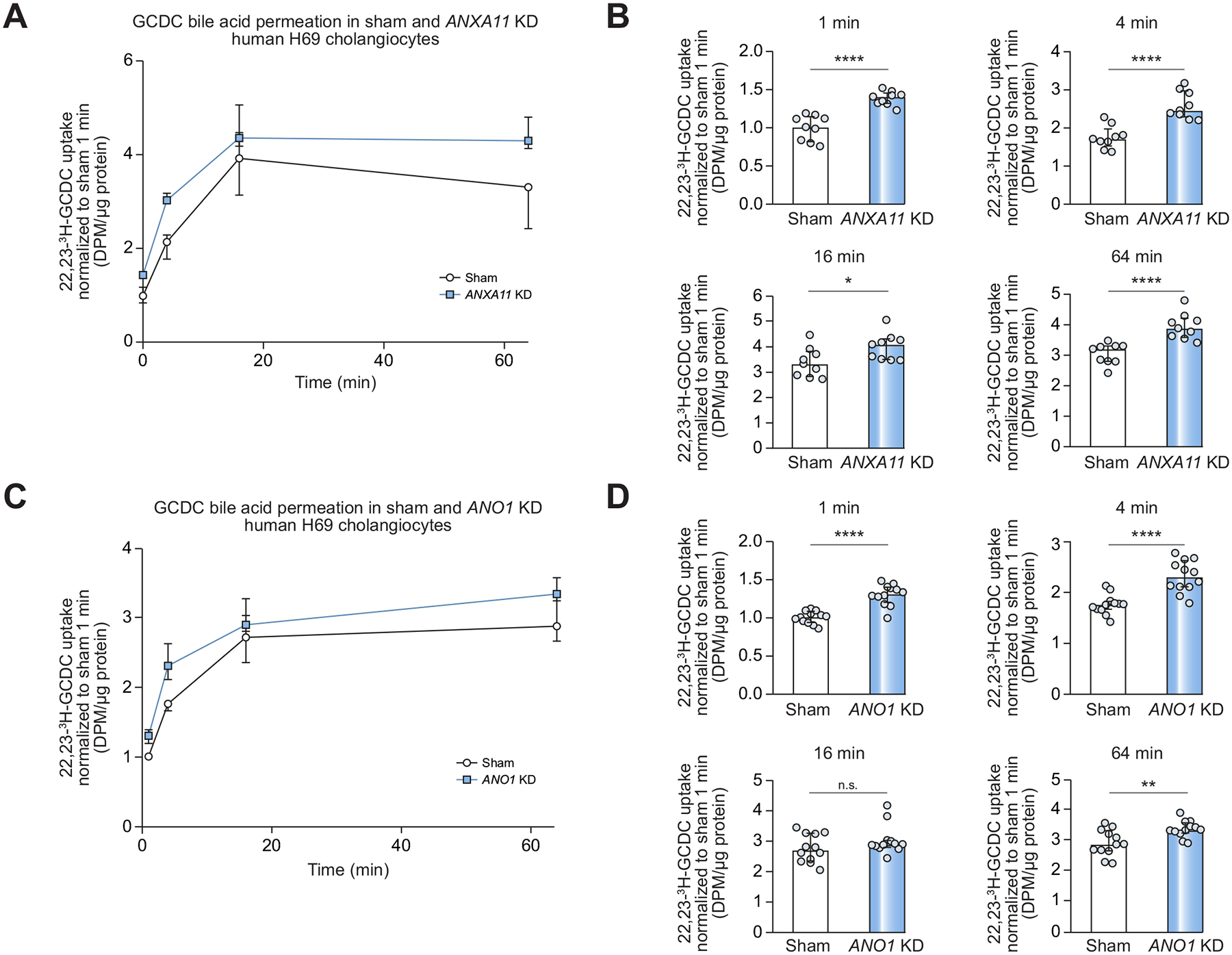
Knockdown of *ANXA11* and *ANO1* increases plasma membrane permeability for glycine-conjugated bile acids in H69 cholangiocytes. 22,23-^3^H-GCDC permeation assay in sham and (A) *ANXA11* KD or (C) *ANO1* KD H69 cholangiocytes. (B, D) Quantification of 22,23-^3^H-GCDC permeation (9 cell samples of n = 3 independent experiments (B) or 12 cell samples of n = 4 independent experiments (D)). Data are presented as median with interquartile range. Level of significance: *p <0.05, **p <0.01, ****p <0.0001, n.s. not significant (unpaired *t* test). DPM, disintegrations per minute; GCDC, glycine-conjugated chenodeoxycholic acid; KD, knockdown.

As ANO1 is crucial to maintain a protective HCO_3_^−^ umbrella on the apical surface of human cholangiocytes,^24,25^ we hypothesized that annexin A11 (as a regulator of ANO1 plasma membrane expression) is needed for a functional HCO_3_^−^ umbrella. Therefore, sham and *ANXA11* KD H69 cells were incubated with 22,23-^3^H-GCDC and bile acid permeation was quantified after 1, 4, 16 and 64 minutes by liquid scintillation counting. 22,23-^3^H-GCDC permeation was markedly increased in *ANXA11* KD H69 cholangiocytes at all time points compared to sham cells (Fig. 6A, B). To confirm this observation, we repeated the experiment in sham and *ANO1* KD H69 cells. In line with the results in *ANXA11* KD H69 cholangiocytes, 22,23-^3^H-GCDC permeation was significantly increased after 1, 4 and 64 minutes and showed a similar tendency after 16 minutes in *ANO1* KD H69 cholangiocytes compared to the corresponding sham cells (Fig. 6C, D).

Annexin A11 is known to be involved in the intrinsic pathway of cellular apoptosis,^8,9,49^ which we confirmed in H69 cholangiocytes (Fig. S6). We were thus unable to unveil increased GCDC-induced apoptosis as a highly probable consequence of increased GCDC uptake in *ANXA11* KD H69 cholangiocytes using effector caspase-3/7 and metabolic activity as readouts after GCDC stimulation (Fig. S7). Notably, *ANO1* KD cells that were exposed to GCDC showed more apoptosis compared to sham H69 cells (Fig. S8).

In conclusion, the increased GCDC permeation confirms a dysfunctional HCO_3_^−^ umbrella in *ANXA11* KD and *ANO1* KD H69 cholangiocytes.

## Discussion

The present study unraveled the role of annexin A11, previously identified by our group as the first IgG1/IgG4-specific autoantigen in IRC,^7^ in the regulation of apical plasma membrane expression of the Cl^−^ channel ANO1 (TMEM16A) in human cholangiocytes. Our data suggest that cholangiocellular annexin A11 and ANO1 may be dysfunctional in patients with IRC positive for annexin A11 IgG1/IgG4 antibodies due to an IgG1/IgG4-mediated autoreactivity against annexin A11. This would weaken the cholangiocyte defense against potentially toxic biliary bile acids, coined the ‘biliary HCO_3_^−^ umbrella’^25–27^ and, thereby, may contribute to the pathogenesis of IRC.

The following observations support the critical role of annexin A11 as a regulator of ANO1 plasma membrane localization: (i) KD of annexin A11 expression in human H69 cholangiocytes resulted in a ~85% decrease in plasma membrane localization of ANO1. ANO1 protein levels were also reduced in the whole cell lysate of *ANXA11* KD H69 cholangiocytes. This is likely a result of the impaired plasma membrane insertion, as untargeted plasma membrane proteins in the endocytic compartment are subjected to rapid degradation.^50^ Notably, in contrast to ANO1, the plasma membrane localization of AE2 was not affected by *ANXA11* KD. (ii) Annexin A11 and ANO1 colocalize in human bile ducts and show a distinct clustering. (iii) Colocalization of annexin A11 and ANO1 in human H69 cholangiocytes depends on intracellular free Ca^2+^ levels. Trafficking of ANO1 to the apical cell membrane is likely Ca^2+^-dependent.^23^ Based on our observations, it is tempting to discuss potential mechanisms for the regulation of ANO1 plasma membrane localization. Annexin A11 was shown to mediate an early step in vesicle trafficking from the ER to the trans-Golgi network.^18^ Indeed, following a rise in intracellular free Ca^2+^ levels, annexin A11 colocalized with ANO1 in vesicular structures in the cytosol (partly close to the nucleus). However, the strongest colocalization was observed at the plasma membrane, which rather favors a late exocytotic step at or close to the apical plasma membrane. Although colocalization suggests a close proximity of annexin A11 with ANO1 and AE2 at the apical plasma membrane,^47,48^ annexin A11 only mediates ANO1 but not AE2 surface expression under the experimental conditions chosen. ANO1 and AE2 likely translocate from the trans-Golgi network to the apical plasma membrane in separate vesicles in cholangiocytes.^51^ In conclusion, annexin A11 regulates the apical plasma membrane localization of ANO1 in human cholangiocytes, probably by mediating Ca^2+^-dependent trafficking or membrane insertion of ANO1.

Based on the regulation of ANO1 plasma membrane localization, this study demonstrated a potential relevance of an IgG1/IgG4-mediated autoreactivity against annexin A11 for the pathogenesis of IRC: (i) Immunohistochemical staining of human liver tissue for annexin A11 showed strong expression of annexin A11 in cholangiocytes, the cell type that is mainly affected in IRC, and weak expression (at most) in hepatocytes. (ii) The predicted epitope sites of anti-annexin A11 IgG1/IgG4-autoantibodies found in the serum of patients with IRC are closely associated with the Ca^2+^ binding domains of annexin A11. (iii) Incubation of human H69 cholangiocytes with IRC patient serum containing high titers of anti-annexin A11 autoantibodies inhibited the Ca^2+^-dependent plasma membrane shift of annexin A11 (a crucial step to exert its function^11^) and decreased the plasma membrane localization of ANO1. Therefore, we hypothesize that autoantibody binding to annexin A11 in patients with IRC might impair Ca^2+^ binding to annexin A11 and, thereby, inhibit Ca^2+^-dependent membrane translocation of annexin A11 and ANO1 trafficking to the apical plasma membrane. This hypothetical pathomechanism would be in line with findings in neurons, where mutations that inhibit Ca^2+^ binding to annexin A11 have been shown to result in both an impaired membrane translocation of annexin A11 and a defective transport function of annexin A11.^11^ The autoantibody-mediated inhibition of annexin A11 membrane translocation most likely requires a cytosolic localization of autoantibody-annexin A11 complexes. Although autoantibodies are believed to not directly permeate the plasma membrane, there is evidence for the presence of autoantibody-antigen complexes in the cytosol and nucleus, which probably contribute to the pathogenesis of autoimmune-mediated diseases such as multiple sclerosis, Sjogren syndrome and systemic lupus erythematosus.^52–56^ The internalization of plasma membrane-bound autoantibody-antigen complexes followed by endosomal leakage is a proposed mechanism of cytosolic entry.^52,54,57^ Whether a similar mechanism applies for autoantibody-annexin A11 complexes in IRC (annexin A11 is potentially accessible for IgG1 and IgG4 autoantibodies on the basolateral surface of human cholangiocytes) remains speculative. (iv) KD of annexin A11 and ANO1 expression in human H69 cholangiocytes increased the plasma membrane permeability for potentially harmful glycine-conjugated bile acids. In human cholangiocytes, ANO1 functions as critical channel for Cl^−^ secretion into the bile duct lumen.^24^ The ANO1-created Cl^−^ gradient is crucial for the transporter AE2 to exchange Cl^−^ for HCO_3_^−^. ANO1 is therefore indispensable for the formation of a protective HCO_3_^−^ umbrella on the apical surface of human cholangiocytes.^25–27^ Glycine-conjugated bile acid influx as a result of an impaired apical biliary HCO_3_- umbrella has been shown to induce cholangiocyte damage,^26,27^ which probably contributes to the progressive bile duct destruction found in immune-mediated cholangiopathies such as PBC and PSC.^28–30,33,34,58,59^ Loss of expression of ANO1 resulted in increased apoptosis in H69 cholangiocytes when exposed to glycine-conjugated chenodeoxycholic acid. Thus, an impaired plasma membrane localization of ANO1 due to autoantibody-mediated inhibition of annexin A11 might result in a defective apical biliary HCO_3_− umbrella, which potentially leads to progressive cholangiocyte damage – a characteristic histopathological finding in IRC.^6^

Based on our present findings on the role of annexin A11 in human cholangiocytes and the functional effects of anti-annexin A11 autoantibodies, we propose the following model: binding of IgG1/IgG4-autoantibodies to annexin A11 might interact with the Ca^2+^ binding sites of annexin A11. As a consequence, annexin A11 may no longer be able to promote Ca^2+^-dependent apical plasma membrane trafficking of ANO1. Reduced membrane protein levels of ANO1 might weaken the biliary HCO_3_− umbrella which in turn could result in bile acid-induced cholangiocyte damage and, thereby, contribute to the pathogenesis of IRC. Of note, the expression of annexin A11 in other organs commonly affected by IgG4-related disease (such as pancreas, salivary glands, prostate) shows a similar expression pattern to that observed in the liver: a compelling predominance of annexin A11 expression in the epithelial HCO_3_^−^-secreting cells targeted by the disease.^10^ This observation, together with the present study, provides a rationale to further investigate the role of annexin A11 and the HCO_3_^−^ umbrella in the pathogenesis of systemic IgG4-related disease.

In conclusion, the IRC autoantigen annexin A11 mediates the apical plasma membrane insertion of the Cl^−^ channel ANO1 in human cholangiocytes, which is crucial for a stable biliary HCO_3_^−^ umbrella. IgG1/IgG4-mediated autoreactivity against annexin A11 may contribute to the pathogenesis of IRC by weakening the biliary HCO_3_^−^ umbrella. IRC might, therefore, represent the third immune-mediated cholangiopathy, next to PBC and PSC,^25–34^ with an underlying secretory defect destabilizing the biliary HCO_3_^−^ umbrella. The role of annexin A11 in other secretory organs targeted by IgG4-related systemic disease remains to be elucidated.

## Supplementary Material

Supplementary Data

Video S1

CTAT Methods

ICMJE Disclosure Form

## Figures and Tables

**Table 1.** Demographic characteristics and laboratory parameters of the patient(s) with IRC and PSC included in the serum treatment experiments.

Patient	Sex (age)	Bilirubin [μmol/L] (0–17)	AP [U/L] (40–120)	GGT [U/L] (0–40)	ALT [U/L] (0–34)	AST [U/L] (0–40)	IgG4 [g/L] (0.08–1.4)	IgG1 reactivity against annexin A11 on western blot	IgG4 reactivity against annexin A11 on western blot
IRC	Male (76)	7	110	67	21	28	5.03	++	++
PSC 1	Female (66)	19	140	16	24	36	n.d.	−	−
PSC 2	Female (60)	9	162	54	28	29	n.d.	−	−

Normal ranges are shown in brackets. (−) not detectable, (++) detectable (high titers). IgG1/IgG4 reactivity against annexin A11 was tested in a previous study.^7^

ALT, alanine aminotransferase; AP, alkaline phosphatase; AST, aspartate aminotransferase; GGT, gamma-glutamyltransferase; IRC, IgG4-related cholangitis; PSC, primary sclerosing cholangitis; n.d., not determined.

## Data Availability

The data that support the findings of this study are included within the article and its supplementary materials.

## Highlights

- Annexin A11 is strongly expressed in human cholangiocytes, but not hepatocytes.
- Annexin A11 mediates plasma membrane expression of the chloride channel ANO1.
- Binding of autoantibodies in IgG4-related cholangitis impairs annexin A11 function.
- Knockdown of *ANXA11* in cholangiocytes destabilizes the ‘biliary HCO_3_^−^ umbrella’.
- A defective HCO_3_^−^ umbrella may foster biliary injury in IgG4-related cholangitis.

## Abbreviations

AE2: anion exchanger 2
ANO1: anoctamin-1
BCECF: 2’,7’-Bis-(2-carboxyethyl)-5-(and-6)-carboxyfluorescein
ER: endoplasmic reticulum
GCDC: glycochenodeoxycholate
HRP: horseradish peroxidase
IRC: IgG4-related cholangitis
PBC: primary biliary cholangitis
PSC: primary sclerosing cholangitis
qRT-PCR: quantitative reverse-transcription PCR
RIPA: radioimmunoprecipitation assay
ROI: region of interest
shRNA: short hairpin RNA
TBST: tris-buffered saline with 0.05% (w/v) Tween 20
TMEM16A: transmembrane member 16 A
