# Supplementary material for: Role of the IgG4-related cholangitis autoantigen annexin A11 in cholangiocyte protection
Source: J Hepatol. Author manuscript; Available in PMC 2024 Jan 23. (PMC10804347; doi:10.1016/j.jhep.2021.10.009)
Supplement: Supplementary Data [file NIHMS1956559-supplement-Supplementary_Data.pdf]

Role of the IgG4-related cholangitis autoantigen annexin A11 in cholangiocyte protection

Toni Herta, Remco Kersten, Jung-Chin Chang, Lowiek Hubers, Simei Go, Dagmar Tolenaars, Coen C. Paulusma, Michael H. Nathanson, Ronald Oude Elferink, Stan F.J. van de Graaf, Ulrich Beuers

Table of contents

Supplementary methods……………………………………………………………………...2

Fig. S1…………………………………………………………......................……………...4

Fig. S2……………………………………………………………….....................……...5

Fig. S3……………………………………………………………….....................……...6

Fig. S4…………………………………………………………......................……………...7

Fig. S5………………………………………………………......................………………...8

Fig. S6……………………………………………………………......................…………...9

Fig. S7…………………………………………………………......................……………10

Fig. S8………………………………………………………………….......................…11

Table S1…………………………………………………………….....................………...12

Table S2…………………………………………………………….....................……...13

Table S3………………………………………………………….....................…………...14

Supplementary references……………………………………………......……………....15

Supplementary methods

Culture of primary mouse hepatocytes

Primary mouse hepatocytes, isolated from C57BL/6 mice (obtained from Envigo; animal experiments were approved by the institutional animal experiment committee), were cultured in 6-well plates (VWR Radnor, PA) precoated with 100 µl per well of 1.5 mg/ml (pH 7.4) type I rat-tail collagen (BD Biosciences, Franklin Lakes, NJ) in a 5% CO_2 incubator as previously described [1] and were passaged twice per week. The culture medium contained Dulbecco's modified Eagle's medium (Life Technologies, Carlsbad, CA) supplemented with 4.5 g/l glucose, 1 mM L-glutamine, 37.5 U/ml penicillin, 37.5 µg/ml streptomycin, 1.75 g/l sodium bicarbonate, 20 mM 4-(2-hydroxyethyl)-1-piperazine ethanesulfonic acid (pH 7.4), and 5% fetal bovine serum (Thermo Scientific, Waltham, MS).

Culture of primary mouse cholangiocytes

Primary mouse cholangiocytes, isolated from C57BL/6 mice (see above), were cultured in 12-well plates (VWR, Radnor, PA) precoated with 1 ml per well of 1.5 mg/ml (pH 7.4) type I rat-tail collagen (Corning, Corning, United Kingdom) in a 5% CO_2 incubator as previously described [2] and were passaged twice per week. The full hormone-supplemented culture medium contained all components as detailed in [3].

Caspase-3/7 activity assay

Confluent H69 cholangiocyte monolayers in 96-well plates were treated with Raptinal (Biovision, Milpitas, CA) in H69 culture medium pH 7.4 for 90 minutes or sodium glycochenodeoxycholate (GCDC, Sigma-Aldrich,St. Louis, MO) or Raptinal in H69 culture medium pH 7.4, pH 6.9 or pH

6.4 (titrated with 1 M HCl) for 4 hours at 37 °C and 5% CO_2 at the indicated concentrations. Apoptosis was quantified by effector caspase-3/7 activity assay (AnaSpec,Fremont, CA) according to manufacturer's instructions. Kinetics of fluorophore caspase-3/7 cleaved Rh110 release was measured by NOVOstar microplate reader (BMG Labtech GmbH, Offenburg, Germany) or CLARIOstar microplate reader (BMG Labtech GmbH, Offenburg, Germany) at λ_{ex} / λ_{em} = 480 / 510 nm. The slope in the linear window of the measured kinetics was calculated and defined as caspase-3/7 activity.

WST-1 metabolic activity assay

Metabolic activity as indicator of cell viability was determined by conversion of WST-1 reagent (Roche Diagnostics, Rotkreuz, Switzerland) to formazan. Confluent H69 cholangiocyte monolayers in 96-well plates were treated with sodium glycochenodeoxycholate (GCDC, Sigma-Aldrich, St. Louis, MO) or Raptinal (Biovision, Milpitas, CA) in H69 culture medium pH 7.4 or pH 6.4 (titraded with 1 M HCl) for 4 hours at 37 °C and 5% CO_2. After treatment, monolayers were washed with H69 culture medium and incubated with WST-1 (1:10 dilution in H69 culture medium). Absorbance at 450 nm (formazan) and 690 nm (reference) was measured after adding of WST-1 (timepoint 0 minutes) and after 30 minutes of incubation at 37°C and 5% CO_2 using Synergy HT multi-mode microplate reader (BioTek, Winooski, VT). The conversion rate (metabolic activity) was calculated from the slope.

Supplementary Figures

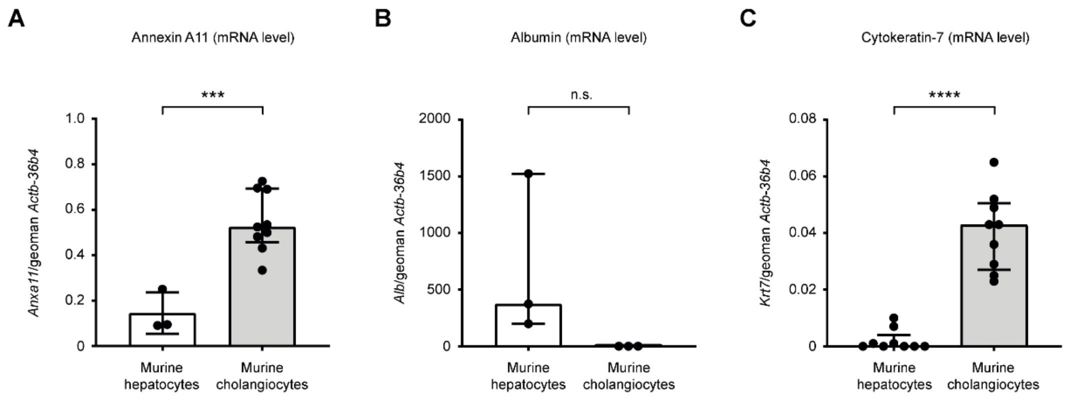

Fig. S1: Expression of *Anxa11*, *Alb* and *Krt7* in murine hepatocytes and cholangiocytes. Expression of *Anxa11*, *Alb* and *Krt7* in primary mouse hepatocytes and cholangiocytes was analyzed using quantitative reverse-transcription polymerase chain reaction (qPCR). (A) *Anxa11* was strongly expressed in murine cholangiocytes but weakly in murine hepatocytes. Expression of (B) *Alb* and (C) *Krt7* served as cell-specific markers for murine hepatocytes (*Alb*) and murine cholangiocytes (*Krt7*). Data are presented as median with interquartile range (3 hepatocyte samples and 9 cholangiocyte samples of n=3 independent experiments (A), 3 hepatocyte samples and 3 cholangiocyte samples of n=3 independent experiments (B), 9 hepatocyte samples and 9 cholangiocyte samples of n=3 independent experiments (C)). Levels of significance: *** $p<0.001$, **** $p<0.0001$, n.s. not significant (unpaired t-test).

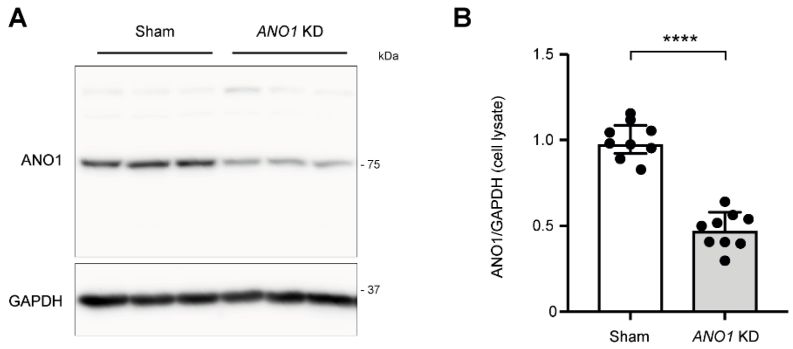

Fig. S2: Validation of *ANO1* knockdown in human H69 cholangiocytes. H69 cholangiocytes were transduced with non-targeting shRNA (sham) or shRNA against *ANO1* (*ANO1* KD). (A) Expression of ANO1 was analyzed in total cell lysates using immunoblotting. ANO1 expression was reduced by ~50% in *ANO1* KD compared to sham transduced H69 cholangiocytes. (B) The densitometries of the ANO1 and GAPDH bands were quantified using ImageJ. The densitometry ratios of ANO1 to GAPDH were calculated and displayed relative to sham H69 cholangiocytes. Data are presented as median with interquartile range (9 cell samples of n=3 independent experiments). Level of significance: **** p<0.0001 (Mann-Whitney U test). ANO1, anoctamin-1; GAPDH, glyceraldehyde 3-phosphate dehydrogenase.

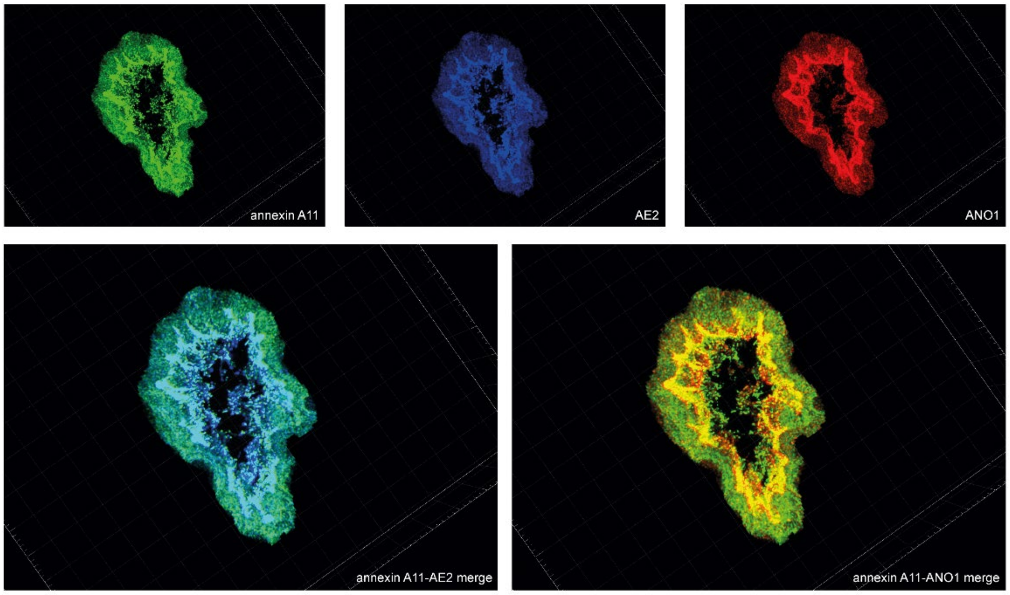

Fig. S3: Apical membrane region of interest of human cholangiocytes used for the calculation of Pearson's correlation coefficients. Series of z-stack confocal microscopy images of the apical region of interest (ROI) were deconvoluted using Huygens Deconvolution Professional software. Shown here is a representative sample of 7 intrahepatic human bile ducts stained for annexin A11 (green), AE2 (blue) and ANO1 (red) and the colocalization of annexin A11 with AE2 and ANO1, respectively. Colocalization between annexin A11 and AE2 and annexin A11 and ANO1 was assessed by their Pearson's correlation coefficients. All samples were included in the calculation of the coefficients. AE2, anion exchanger 2; ANO1, anoctamin-1.

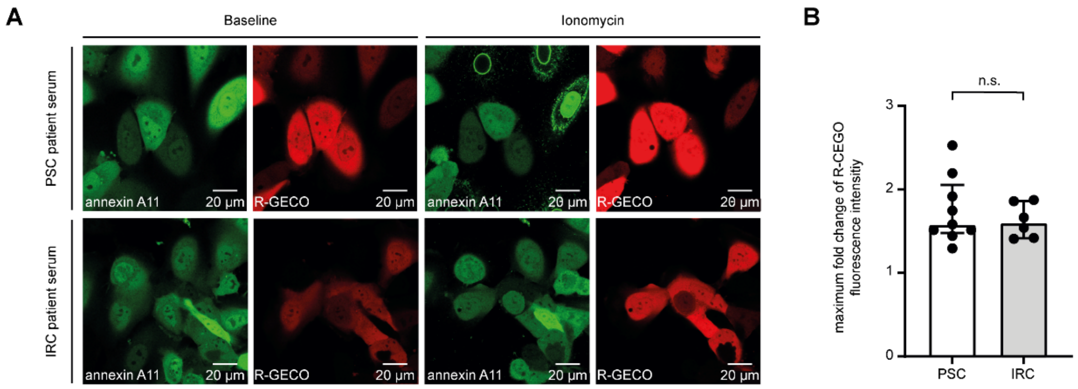

Fig. S4: Ionomycin-induced fold-change of R-GECO1 maximum fluorescence intensity in annexin A11-mEmerald H69 cholangiocytes after incubation with PSC or IRC patient serum. (A) Annexin A11-mEmerald H69 cholangiocytes were transiently transfected with the Ca^{2+} indicator R-GECO1 and incubated for 3 days with 20% PSC (without anti-annexin A11 autoantibodies) or IRC patient serum (with anti-annexin A11 autoantibodies). Cells were imaged before (baseline) and after 15 minutes of incubation with 50 µM ionomycine. A strong membrane shift of annexin A11-mEmerald (green) was observed after incubation with PSC but not IRC patient serum. Maximum fold-change of R-GECO1 fluorescence (red) did not differ in PSC and IRC serum incubated H69 cholangiocytes. (B) Maximum fold-change of R-GECO1 fluorescence intensity relative to baseline was quantified in R-GECO1 transfected annexin A11-mEmerald H69 cholangiocytes (region of interest, ROI) using ImageJ. Data are presented as median with interquartile range (9 confocal pictures of PSC serum incubation and 6 confocal pictures of IRC serum incubation of n=3 independent experiments). Level of significance: n.s. not significant (unpaired t-test). IRC, immunoglobulin G4-related cholangitis; PSC, primary sclerosing cholangitis.

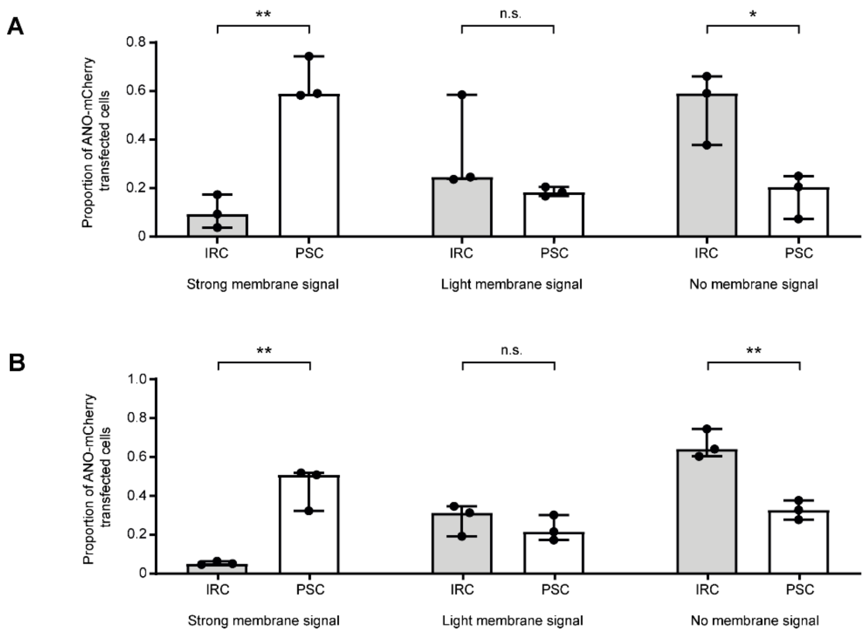

Fig. S5: ANO1 plasma membrane localization in H69 cholangiocytes after incubation with IRC or PSC patient serum. H69 cholangiocytes were transiently transfected with ANO1-mCherry and incubated for 6 days with 20% IRC patient serum (with anti-annexin A11 autoantibodies) or PSC patient serum (without anti-annexin A11 autoantibodies). Semiquantitative scoring of ANO1-mCherry plasma membrane staining was done separately in a blinded fashion by 3 researchers. (A) and (B) shows the separate scoring of n=3 independent experiments performed by 2 researchers (see Figure 5D for the third researcher). The majority of cells showed a strong plasma membrane localization of ANO1-mCherry after incubation with PSC patient serum but no to light plasma membrane localization of ANO1-mCherry after incubation with IRC patient serum. Data are presented as median with interquartile range. Level of significance: * $p<0.05$, ** $p<0.01$, n.s. not significant (two-way ANOVA). IRC, immunoglobulin G4-related cholangitis; PSC, primary sclerosing cholangitis.

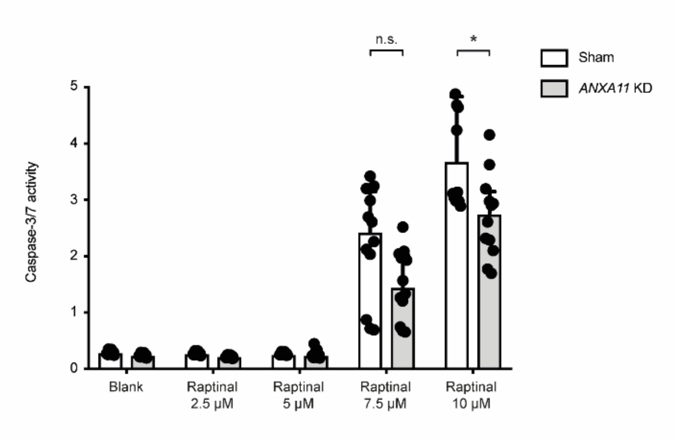

Fig. S6: Annexin A11 mediates Raptinal-induced apoptosis in human cholangiocytes. Determination of caspase-3/7 activity in sham and *ANXA11* KD H69 cholangiocytes after stimulation with 2.5, 5, 7.5 or 10 µM Raptinal (activator of intrinsic apoptosis pathway) at pH 7.4 for 90 minutes. Induction of effector caspase-3/7 activity was reduced in *ANXA11* KD H69 cells compared to sham transduced H69 cells after stimulation with 10 µM Raptinal. Data are presented as median with interquartile range (12 cell samples of n=4 independent experiments). Level of significance: * $p<0.05$, n.s. not significant (unpaired t-test).

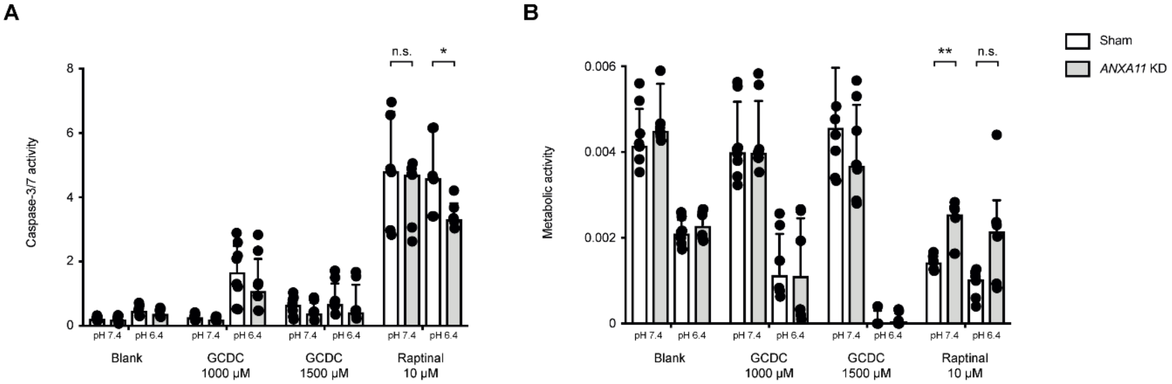

Fig. S7: Caspase-3/7 activity and cell viability in sham transduced and *ANXA11* KD H69 cholangiocytes after stimulation with GCDC. (A) Caspase-3/7 activity and (B) metabolic activity in sham and *ANXA11* KD H69 cholangiocytes after bile salt stimulation with 1000 or 1500 µM glycine-conjugated chenodeoxycholic acid (GCDC) or stimulation with 10 µM Raptinal at pH 7.4 and pH 6.4 for 4 hours. *ANXA11* KD reduced induction of caspase-3/7 activity and increased metabolic activity in response to Raptinal but not to GCDC at pH 7.4 and 6.4. Data are presented as median with interquartile range (8 cell samples of GCDC stimulation and 6 cell samples of Raptinal stimulation of n=3 independent experiments). Level of significance: * p<0.05, ** p<0.01, n.s. not significant (unpaired t-test).

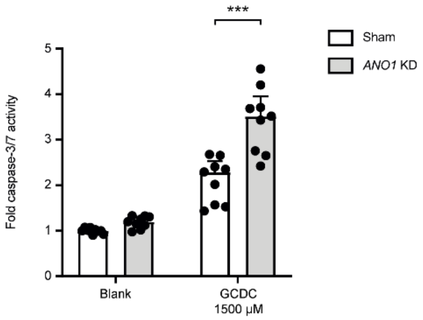

Fig. S8: ANO1-deficient human cholangiocytes are sensitive to bile acid-induced apoptosis. Caspase-3/7 activity was determined in sham transduced and *ANO1* KD H69 cholangiocytes after stimulation with 1500 µM GCDC at pH 6.9 for 4 hours. *ANO1* KD increased induction of caspase-3/7 activity in response to GCDC. Values were corrected for total protein content per well and are shown as fold-change relative to unstimulated sham H69 cholangiocytes. Data are presented as median with interquartile range (9 cell samples of n=3 independent experiments). Level of significance: *** $p<0.001$ (unpaired t-test).

Supplementary tables

Table S1: primary and secondary antibodies used for immunoblotting

Antibody	Company	Catalog No	Dilution
Anti-annexin A11	Santa Cruz	Sc-46686	1:1000
Anti-Na^+/K^+-ATPase	Gift from Dr. Jan B. Koenderink		1:5000
Anti-GAPDH	Cell Signaling	14C10	1:5000
Anti-AE2	Santa Cruz	Sc-376632	1:1000
Anti-ANO1 C5	Santa Cruz	Sc-377115	1:1000
Anti-Goat IgG	Dakoplatt	PO160	1:5000
Anti-Rabbit IgG	BioRad	170-6515	1:5000
Anti-Mouse IgG	BioRad	170-6516	1:5000

Table S2: formulation of normal and chloride-free HBSS for 5% CO2

Compound	Normal HBSS for 5% CO2	Chloride-free HBSS for 5% CO2
NaCl	119.78 mM	-
Na gluconate	-	119.88 mM
$CaCl_2$	1.27 mM	-
Ca gluconate	-	1.27 mM
KCl	5.37 mM	-
K gluconate	-	5.37 mM
KH_2PO_4	0.44 mM	0.44 mM
Na_2HPO_4	0.34 mM	0.34 mM
$MgSO_4$	0.81 mM	0.81 mM
$NaHCO_3$	21.43 mM	21.43 mM
Glucose	5.55 mM	5.55 mM
HEPES-NaOH, pH 7.4	20 mM	20 mM

Table S3: primer sequences for quantitative reverse-transcription PCR

Target	Forward primer (5' to 3')	Reverse primer (5' to 3')
Human *ANXA11*	GGCTTACGGCAAGGATTTGA	CGGGAAGCGAGGATCTCAAT
Human *ACTB*	AGAGCTACGAGCTGCCTGAC	AGCACTGTGTTGGCGTACAG
Human *36B4*	TCATCAACGGGTACAAACGA	GCCTTGACCTTTTCAGCAAG
Mouse *Anxa11*	GGCAAGTCACTGTACCACGA	AGTGTGCTCTTTGGGACGTT
Mouse *Actb*	TTCTTTGCAGCTCCTTCGTT	ATGGAGGGGAATACAGCCC
Mouse *36b4*	CCAGCGAGGCCACACTGCTG	ACACTGGCCACGTTGCGGAC

Supplementary references

1. Gilglioni, E.H., et al., *Improved oxygenation dramatically alters metabolism and gene expression in cultured primary mouse hepatocytes.* Hepatology Communications, 2018. **2**(3): p. 299-312.
2. Chang, J.C., et al., *Soluble Adenylyl Cyclase Regulates Bile Salt-Induced Apoptosis in Human Cholangiocytes.* Hepatology, 2016. **64**(2): p. 522-34.
3. Vroman, B. and N.F. LaRusso, *Development and characterization of polarized primary cultures of rat intrahepatic bile duct epithelial cells.* Lab Invest, 1996. **74**(1): p. 303-13.
