# Supplementary material for: Role of the IgG4-related cholangitis autoantigen annexin A11 in cholangiocyte protection
Source: J Hepatol. Author manuscript; Available in PMC 2024 Jan 23. (PMC10804347; doi:10.1016/j.jhep.2021.10.009)
Supplement: CTAT Methods [file NIHMS1956559-supplement-CTAT_Methods.docx]

**Journal of Hepatology**

**CTAT methods**

Tables for a “Complete, Transparent, Accurate and Timely account” (CTAT) are now mandatory for all revised submissions. The aim is to enhance the reproducibility of methods.

- Only include the parts relevant to your study
- Refer to the CTAT in the main text as ‘Supplementary CTAT Table’
- Do not add subheadings
- Add as many rows as needed to include all information
- Only include one item per row

**If the CTAT form is not relevant to your study, please outline the reasons why:**

|  |
| --- |

- 1. **Antibodies**

| **Name** | **Citation** | **Supplier** | **Cat no.** | **Clone no.** |
| --- | --- | --- | --- | --- |
| Annexin A11 | No citation | Santa Cruz | Sc-46686 | D-12 |
| Na^+^/K^+^-ATPase | Koenderink JB et al., *J Biol Chem* 2003; 278(51):51213-22 | Gift from Dr. Jan B. Koenderink |  |  |
| GAPDH | Privitera L et al., *J Neurosci* 2020; 40(24):4644-60 | Cell Signaling | 14C10 |  |
| AE2 | Eladari D et al., *Am J Physiol* 1998; 275:334-42 | Santa Cruz | Sc-376632 | D-3 |
| AE2 | Liu L et al., *J Cell Physiol* 2011; 226(6):1702-12 | Santa Cruz | Sc-46710 | N-12 |
| ANO1 | Jiang Y et al., *Oncol Rep* 2019; 41(4):2361-70 | Santa Cruz | Sc-377115 | C-5 |
| ANO1 | Lv XF et al., *Theranostics* 2020; 10(9):3980-93 | Abcam | ab53212 |  |
| Rabbit anti-goat IgG HRP-conjugated | Waasdorp M et al., *J Cell Mol Med* 2019; 23(2):1268-79 | Dako | P0160 |  |
| Goat anti-rabbit IgG HRP-conjugated | Vitor AC et al., *Sci Adv* 2019; 5(1):eaau1249 | BioRad | 170-6515 |  |
| Goat anti-mouse IgG HRP-conjugated | Roof AK et al., *Endocrinology* 2018; 159(6):2421-34 | BioRad | 170-6516 |  |
| Rabbit anti-goat IgG AP-conjugated | Pivarsci A et al., *Int Immunol* 2003; 15(6):721-30 | Sigma | A4187 |  |
| Donkey anti-goat IgG Alexa Fluor 568 | Nelson AR et al., *Front Aging Neurosci* 2020; 12:108 | Invitrogen | A-11057 |  |
| Goat anti-rabbit Alexa Fluor 555 | Saju JM et al., *Cell Rep* 2018; 25(12):3252-61 | Invitrogen | A27039 |  |
| Goat anti-mouse Alexa Fluor 488 | Danziger O et al., *Oncotarget* 2016; 7(32):52115-34 | Invitrogen | A28175 |  |
| Donkey anti-goat Alexa Fluor 647 | Fan X et al., *Nat Commun* 2019; 10(1):3164 | Invitrogen | A21447 |  |

- 1. **Cell lines**

| **Name** | **Citation** | **Supplier** | **Cat no.** | **Passage no.** | **Authentication test method** |
| --- | --- | --- | --- | --- | --- |
| H69 cells | Grubman SA et al, *Am J Physiol* 1994; 266:G1060-G1070 | Gift from Dr. Douglas Jefferson |  | P05 to P35 | qPCR for cholangiocyte markers (see sequence based reagents) |

- 1. **Organisms**

| **Name** | **Citation** | **Supplier** | **Strain** | **Sex** | **Age** | **Overall n number** |
| --- | --- | --- | --- | --- | --- | --- |
|  |  |  |  |  |  |  |

- 1. **Sequence based reagents**

| **Name** | **Sequence** | **Supplier** |
| --- | --- | --- |
| h*SOX9* | **Fw:** GGCAAGCTCTGGAGACTTCTG  **Rv:** GTTCTTCACCGACTTCCTCCG | Sigma-Aldrich |
| h*GGT1* | **Fw:** TGCTCGAAGATTGGGAGGGA  **Rv:** TGATGACCTCAGCTTTTCGTGT | Sigma-Aldrich |
| h*AQP1* | **Fw:** AAGTTGCTCACCGACTCACC  **Rv:** CAAGCACCATTGGGAGCAAG | Sigma-Aldrich |
| h*KRT19* | **Fw:** GTCACAGCTGAGCATGAAAGC  **Rv:** GCTCACTATCAGCTCGCACA | Sigma-Aldrich |
| h*GPBAR1* | **Fw:** GTCCTGCCTCCTCGTCTACT  **Rv:** GCCTCAGGACTGCCATGTAG | Sigma-Aldrich |
| h*ITPR3* | **Fw:** GACTTCCTGCTCCTTCTACG  **Rv:** CATTTCACTCATGGCTGCGG | Sigma-Aldrich |
| h*ANXA11* | **Fw:** GGCTTACGGCAAGGATTTGA  **Rv:** CGGGAAGCGAGGATCTCAAT | Sigma-Aldrich |
| h*SLC4A2* | **Fw:** GTCACAGCTGAGCATGAAAGC  **Rv:** GCTCACTATCAGCTCGCACA | Sigma-Aldrich |
| h*ANO1* | **Fw:** TCACCAAGATCGAGGTCCCA  **Rv:** AATGTACACGTAGTCGCCCG | Sigma-Aldrich |
| h*CAII* | **Fw:** TTACTGGACCTACCCAGGCT  **Rv:** ACGGAATTTCAACACCTGCTC | Sigma-Aldrich |
| h*CAIX* | **Fw:** TACAGCTGAACTTCCGAGCG  **Rv:** AATTCAGCTGGACTGGCTCA | Sigma-Aldrich |
| h*ACTB* | **Fw:** AGAGCTACGAGCTGCCTGAC  **Rv:** AGCACTGTGTTGGCGTACAG | Sigma-Aldrich |
| h*36B4* | **Fw:** TCATCAACGGGTACAAACGA  **Rv:** GCCTTGACCTTTTCAGCAAG | Sigma-Aldrich |
| m*Anxa11* | **Fw:** GGCAAGTCACTGTACCACGA  **Rv:** AGTGTGCTCTTTGGGACGTT | Sigma-Aldrich |
| m*Alb* | **Fw:** CAGCGGAGCAACTGAAGACT  **Rv:** AAGGTTTGGACCCTCAGTCG | Sigma-Aldrich |
| m*Krt7* | **Fw:** TCCTTCATCGACAAGGTACGC  **Rv:** CCTCAGCCCAGCAATCTGAG | Sigma-Aldrich |
| mActb | **Fw:** TTCTTTGCAGCTCCTTCGTT  **Rv:** ATGGAGGGGAATACAGCCC | Sigma-Aldrich |
| m*36b4* | **Fw:** CCAGCGAGGCCACACTGCTG  **Rv:** ACACTGGCCACGTTGCGGAC | Sigma-Aldrich |

- 1. **Biological samples**

| **Description** | **Source** | **Identifier** |
| --- | --- | --- |
| Paraffin-embedded human liver sample | Departement of Pathology, Amsterdam University Medical Centers, location AMC, Amsterdam, The Netherlands |  |
| Paraffin-embedded human liver samples | Departement of Pathology, Yale University School of Medicine, New Haven, USA |  |
| Human PSC and IRC serum samples | Department of Hepatology and Gastroenterology, Amsterdam University Medical Centers, location AMC, Amsterdam, The Netherlands |  |

- 1. **Deposited data**

| **Name of repository** | **Identifier** | **Link** |
| --- | --- | --- |
|  |  |  |

- 1. **Software**

| **Software name** | **Manufacturer** | **Version** |
| --- | --- | --- |
| Electronic Lab Notebook (eLab Journal ELN) | Bio-ITech BV |  |
| Huygens Deconvolution Professional software | Scientific Volume Imaging |  |
| IMARIS | Oxford Instruments | 9.3.1 |
| ImageJ | Wayne Rasband | 1.50i |
| GraphPad Prism | GraphPad | 6 |
| LinRegPCR | Amsterdam UMC | 2013.0 |
| ElliPro | Pnomarenko et al. | 1 |
| Jmol | Jmol development team | 14 |
| ANXA11 Rattus norvegicus crystal structure (6TU2) | Raasakka et al. |  |

- 1. **Other (e.g. drugs, proteins, vectors etc.)**

| shRNA ANXA11 | TRCN0000056377 | Sigma-Aldrich |
| --- | --- | --- |
| shRNA ANO1 | TCRN0000040263 | Sigma-Aldrich |
| shRNA control | SHC002 | Sigma-Aldrich |
| Annexin A11-mEmerald | #164210 | Addgene |
| R-GECO1 | #32444 | Addgene |
| ANO1-mCherry | - | Dr. Lily Yeh Jan |
| NHS-ss-Biotin | 21331 | Thermo Scientific |
| Complete^TM^ Protease Inhibitor Cocktail | 11697498001 | Roche |
| Pierce^tm^ BCA Protein Assay Kit | 23225 | Thermo Scientific |
| Neutravidin beads | 29202 | Thermo Scientific |
| Lumi-light | 12015196001 | Roche |
| BCECF-AM | B1170 | Thermo Scientific |
| Trilogy | 920P-04 | Cell Marque |
| ProLong^TM^ Gold Antifade Mountant | P10144 | Thermo Scientific |
| Ionomycin | ab120116 | Abcam |
| Leibowitz phenol red free | 21083027 | Thermo Scientific |
| CellMask Green | C37608 | Thermo Scientific |
| HBSS phenol red free | 10-527F | Lonza |
| Glycochenodeoxycholate | G0759 | Sigma-Aldrich |
| 22,23-^3^H-sodium glycochenodeoxycholate | - | Dr. Alan Hofmann |
| Fatty-acid free BSA | A6003 | Sigma-Aldrich |
| Digitonin | 3043 | Merck |

- 1. **Please provide the details of the corresponding methods author for the manuscript:**

| Toni Herta, Department of Gastroenterology and Hepatology and Tytgat Institute for Liver and Intestinal Research, AGEM, Amsterdam University Medical Centers, location AMC, Amsterdam, The Netherlands, Email: t.herta@amsterdamumc.nl |
| --- |

**2.0 Please confirm for randomised controlled trials all versions of the clinical protocol are included in the submission. These will be published online as supplementary information.**

|  |
| --- |
